# The Chemical, Microbiological and Volatile Composition of Kefir-like Beverages Produced from Red Table Grape Juice in Repeated 24-h Fed-Batch Subcultures

**DOI:** 10.3390/foods11193117

**Published:** 2022-10-07

**Authors:** Delicia L. Bazán, Pablo G. del Río, José Manuel Domínguez, Sandra Cortés-Diéguez, Juan C. Mejuto, Nelson Pérez-Guerra

**Affiliations:** 1 Academic Department of Food Industries Engineering, Engineering Faculty, National University of Jaén, Jr. Cuzco 250, Pueblo Libre, Jaén 06801, Peru; 2 Department of Analytical and Food Chemistry, Faculty of Sciences, University of Vigo, 32004 Ourense, Spain; 3 Department of Chemical Engineering, Faculty of Sciences, University of Vigo, 32004 Ourense, Spain; 4 Industrial Biotechnology and Environmental Engineering Group “BiotecnIA”, Chemical Engineering Department, Faculty of Sciences, University of Vigo, 32004 Ourense, Spain; 5 Department of Physical Chemistry, Faculty of Sciences, University of Vigo, 32004 Ourense, Spain

**Keywords:** kefir grains, grapes, kefir-like beverage, fed-batch fermentation, volatile compounds

## Abstract

The aim of this work was to study the production of kefir-like beverages via the fed-batch fermentation of red table grape juice at initial pHs of 3.99 (fermentation A) and 5.99 (fermentation B) with kefir grains during 4 repeated 24-h fed-batch subcultures. All kefir-like beverages (KLB) were characterized by low alcoholic grade (≤3.6%, *v*/*v*) and lactic and acetic acid concentrations. The beverages obtained from fermentation B had lower concentrations of sugars and higher microbial counts than the KLB obtained in fermentation A. Additionally, the KLB samples from fermentation B were the most aromatic and had the highest contents of alcohols, esters, aldehydes and organic acids, in contrast with the nonfermented juice and KLB from fermentation A. These results indicate the possibility of obtaining red table grape KLB with their own distinctive aromatic characteristics and high content in probiotic viable cells, contributing to the valorization of this fruit.

## 1. Introduction

Today, there is an increasing interest of consumers in healthier ecological and functional foods containing biologically active components due to the development of knowledge about the potential positive health effects produced by these foods [1].

Kefir is a refreshing, creamy and slightly carbonated drink with a low level of ethanol and acetic acid, a slight acid or bitter taste and a mild aroma similar to fresh yeast [2]. This drink is obtained from the fermentation of milk with kefir grains, which mainly contain lactic acid bacteria (LAB), acetic acid bacteria (AAB) and yeasts that grow in symbiosis and are held together by a matrix of proteins and a polysaccharide composed of glucose and galactose units, known as kefiran [3,4]. The final pH of kefir normally ranges between 4.3 and 4.4, due to the production of lactic acid by LAB from lactose. The contents of lactic acid, ethanol and CO_2_ are controlled by the incubation temperature during the production process [5].

Kefir has high nutritional value and health-improving properties that are associated with the presence in this drink of a high concentration of viable probiotic microorganisms (LAB, AAB and yeasts) and fermentation products including kefiran, enzymes (β-galactosidase), organic acids and volatile compounds [6]. According to different researchers, the consumption of kefir: (i) improves the tolerance and digestion of lactose in lactose-intolerant individuals [7]; (ii) helps to treat tuberculosis, obesity, constipation, disease inflammatory bowel disease, allergies and even cancer; and (iii) controls increasing blood pressure and reduces cholesterol levels serum [8].

Recently, different nondairy substrates have been assayed as producing potentially probiotic kefir-like beverages with high contents of probiotic strains, including molasses, coconut [9], cocoa pulp [10], and more recently, juices extracted from carrot, fennel, melon, onion, tomato, strawberry [11], apple, grape, kiwifruit, pomegranate, prickly pear and quince [12].

Grape contains various nutrients such as vitamins, minerals, carbohydrates, edible fibers and phytochemicals. Polyphenols are the most important phytochemicals present in grapes because of their biological activity and potential health effects, including the inhibition of some degenerative diseases such as cardiovascular diseases [13] and some types of cancer [14,15]. These compounds also slow aging [16] and have a preservative effect against the oxidation of food [17].

Considering the beneficial effects of kefir beverages and grapes, the heterogeneity of substrates that can be fermented by the kefir grains and the gradual reduction in the consumption of table grapes in Spain in recent years [18], the use of this fruit as a substrate for obtaining a potentially probiotic beverage is an interesting alternative that could contribute to the valorization of table grapes. In addition, this approach could contribute to increasing the Spanish producers’ incomes since the marketing of fermented beverages from grapes could facilitate the commercialization of kefir-like beverages from red table grapes.

Since in pure cultures of LAB, the use of fed-batch fermentation improved the free biomass production [9,19,20], the use of this fermentation procedure to produce a kefir-like beverage from table grape juice with kefir grains could offer the possibility of obtaining a drink with a high concentration of probiotic cells.

However, there is no information available on the kinetics of the batch or fed-batch fermentation of juice from red table grape with milk kefir grains. In addition, most of the studies dealing with the production of kefir-like beverages focused only on the analysis of the chemical, microbiological and volatile compositions of the drinks obtained after batch fermentation (24, 48 or 72 h or 5 days) with kefir grains or microorganisms isolated from them [1,6,9,10,11,12]. Taking into account these considerations, this work aimed to study the kinetics of the fed-batch fermentation of red table grape juice with kefir grains and characterize both qualitatively and quantitatively the chemical, microbiological and volatile compositions of the beverages obtained. To determine how many subcultures can be made with the activated kefir grains, the production of kefir-like beverages was studied by using the grains as inoculum in four consecutive subcultures. Given the differences in the optimal pH for the growth of LAB, AAB and yeasts present in the kefir grains, 2 fermentations (at initial pH of 3.99 (the native pH of the red table grape juice) and 5.99) were developed to study the effect of this variable on the chemical, microbiological and volatile compound compositions of the kefir-like beverages obtained. With this approach, two different kefir-like beverages were obtained.

## 2. Materials and Methods

### 2.1. Kefir Grains, Activation and Conservation

The milk kefir grains CIDCA AGK1 used in this study were obtained from the Center for Research and Development in Food Cryotechnology (CIDCA, La Plata, Argentina). Before being used as a fermentation entity to produce the kefir-like beverages, the grains were activated with two transfers in pasteurized ultra-high-temperature (UHT) whole milk (Central Lechera Asturiana, Asturias, Spain) with the following composition (g/L), as declared by the producer: pH, 6.75; carbohydrates, 46.0; proteins, 32.0; fats, 36.0; saturated fats, 24.0; salt, 1.0 and calcium, 1.2.

For activation, the kefir grains were separated from the coverage liquid (milk) by filtration through a sterile plastic strainer, weighed (approximately 30.6 g, wet weight) and added to a 1-L Pyrex bottle containing 1 L pasteurized whole milk UHT in a biosafety cabinet. Subsequently, the bottle was covered with a sterile cheesecloth, and it was secured with a rubber band and incubated at room temperature away from direct sunlight at room temperature at 150 rpm for 24 h. Then, the kefir grains were separated from the fermented milk under sterile conditions by filtration through a plastic strainer and washed with sterile distilled water; after the second activation in milk, the grains were used as the inoculum to produce the kefir-like beverages.

The grains were kept at 4 and −20 °C in pasteurized whole milk UHT for storage for short and long times, respectively.

### 2.2. Juice Preparation and Fermentation Conditions

Juice extracted from red table grapes (Red Globe, category I, Peru), obtained from a shopping center (Gadis) in Ourense (Spain), was used as the fermentation substrate. To obtain the juice, the grapes were separated from the clusters and washed three times with sterile distilled water to minimize the presence of traces of possible pesticides used for conservation of the fruit.

Subsequently, the grapes were broken and then squeezed by pressing them in a plastic strainer with the aid of a spatula under sterile conditions to obtain a juice free of skins and seeds. The yield obtained was approximately 366 mL juice per kg of fruit used.

The initial mean composition (g/L) of the fresh juice from red table grape used as culture medium was: glucose, 83.61 ± 4.35; fructose, 101.30 ± 5.23; tartaric acid, 1.50 ± 0.01; malic acid, 0.16 ± 0.01; citric acid, 0.61 ± 0.02; and pH 3.99.

In the present work, two fermentation substrates were used: the grape juice (native pH of 3.99) without pH adjustment and the same medium adjusted to pH 5.99. This was because the pH of the red table grape juice (3.99) is more favorable for the growth of yeasts [21,22] than for the growth of the lactic acid (LAB) and acetic acid bacteria (AAB) present in the kefir grains. In contrast, a pH between 6.0 and 6.5 could be more favorable for the growth of LAB [23,24] and AAB [25] present in the kefir grains.

However, the pH of the table grape juice was not adjusted to 6.0 or above since the red color of the fruit juice abruptly turned bright green when the pH of this substrate changed from 5.99 to 6.00. Considering that this fact could undoubtedly cause a rejection of the potential probiotic beverage by consumers, the juice from red table grapes was adjusted to pH 5.99 with 5 N NaOH to obtain a substrate with a pH more favorable for the growth of LAB and AAB of the kefir grains.

Duplicate fed-batch fermentations were conducted at room temperature and 150 rpm for 24 h, with 4 transfers of the kefir grains in fresh juice, using 250 mL culture bottles containing 100 mL juice at an initial pH of 3.99 (fermentation A) or 5.99 (fermentation B). In the first 24 h cultures, the juices at pH of 3.99 (duplicate 250 mL culture bottles A1) or 5.99 (duplicate 250 mL culture bottles B1) were inoculated with 5 g of the previously activated kefir grains. After 12 h of incubation, the flasks were removed from the incubator, and aliquots of 20 mL of fermented juice of each flask were aseptically taken to determine the concentrations of free biomass, sugars and fermentation products. Then, 20 mL of fresh red table grape juice was added to each flask, and after recording the pH, the flasks were incubated again under the same conditions up to 24 h, when the fermentation was stopped (first subculture).

The fermented media were filtered through a sterile plastic strainer to separate the grains, which were washed with sterile distilled water, air-dried for 10 min in tissue paper in a biosafety cabinet and weighed before their use as inoculum in the corresponding next 24 h culture (duplicate culture bottles A2 and B2). The second (24–48 h), third (48–72 h) and fourth (72–96 h) fed-batch subcultures were carried out under the same conditions as the first subculture.

After separation of the kefir grains, the fermented media obtained at the end of each subculture (24, 48, 72 and 96 h) were divided into 3 aliquots. The 1st aliquot (10 mL) was used to quantify the colony forming units (CFU) of bacteria (rod and mesophilic LAB, total mesophilic, *Enterobacteriaceae*, *Pseudomonas* and AAB) and yeasts.

The 2nd aliquot (80 mL) was centrifuged (5000 rpm/5 min at 4 °C), and the pellets obtained were used to measure the concentration of free biomass in the kefir-like beverages. The resulting supernatant was divided into two subaliquots. The 1st subaliquot (approx. 70 mL) was used to measure the concentrations of volatile compounds. The 2nd subaliquot (10 mL) was used to measure the culture pH and the concentrations of sugars and fermentation products (alcohols and organic acids). After determining the concentrations of biomass (*X*) and products (*P*: lactic acid (LA), acetic acid (AA), succinic acid (SA), malic acid (MA), ethanol (Et) and glycerol (Gly)), the fermentation yield coefficients (*Y*: *Y_X/TSc_*, *Y_LA/TSc_*, *Y_AA/TSc_*, *Y_SA/TSc_*, *Y_MA/TSc_*, *Y_Et/TSc_* and *Y_Gly/TSc_*, in g/g) were calculated as the ratio of the biomass or fermentation product formed (in g) per unit mass of total sugars consumed (*TSc*, in g):

$$Y=\frac{X}{S}\;\mathrm{or}\;Y=\frac{P}{S}$$

The 3rd aliquot (10 mL) was used to determine the antibacterial activity of the beverages. This sample was acidified to pH 3.5 with 5 N HCl (to facilitate the desorption of the bacteriocin molecules from the cell surfaces of the bacteriocin-producing strains [19]), heated in a boiling water bath for 3 min to kill the cells (LAB, AAB and yeasts) and centrifuged (5000 rpm/15 min at 4 °C) to separate dead cells. The cell-free supernatant was adjusted to pH 6.0 with 4 N NaOH to keep the low pH (3.5) of this sample from contributing to the inhibition of the indicator strain in the antibacterial activity assay [19].

### 2.3. Microbiological Counts in the Fermentation Medium

Serial decimal dilutions of samples from the first aliquot were made and plated in triplicate on MRS (de Man, Rogosa and Sharpe) agar (pH 5.4), M17 agar, PCA (plate count agar), double-layered VRBGA (violet red bile glucose agar), PAB (*Pseudomonas* agar base containing 10 g/L cetrimide fucidin, Carr agar, and YEG (yeast extract-glucose) agar for enumeration of rod lactic acid bacteria (LAB), mesophilic LAB, total mesophilic (TM), Enterobacteriaceae, *Pseudomonas* [12], acetic acid bacteria (AAB) [26] and yeasts, respectively. The culture media were purchased from Panreac Quimica SA, Barcelona, Spain (MRS, PCA, PAB and YEG), Difco Laboratories, Detroit, MI, USA (M17), Oxoid, Milan, Italy (VRBGA) and Condalab Laboratories SA, Madrid, Spain (Carr).

Amphotericin B, at a final concentration of 0.1 g/L, was added to the MRS (MRS-A) and Carr (Carr-A) agar media after media sterilization to prevent fungal growth, and chloramphenicol, at a final concentration of 0.1 g/L, was added to YEG (YEG-C) agar after medium sterilization to prevent bacterial growth. The plates were incubated anaerobically at 30 °C for 48 h (MRS-A and M17), or aerobically at 30 °C for 72 h (PCA), 37 °C for 24 h (VRBGA), 20 °C for 48 h (PAB), 30 °C for 48 h (Carr-A) and 25 °C for 48 h (YEG-C). Results (means ± standard deviations of three experiments and two analytical replications each) were expressed as log CFU (colony forming units) per mL fermented juice.

### 2.4. Analytical Methods

The pellets obtained after centrifuging the second aliquot were washed in saline (0.8% (*w*/*v*) NaCl) and centrifuged (5000 rpm/5 min at 4 °C) 2 times, and after resuspending the washed cells again in saline, the optical density (OD) of each sample was measured at 700 nm. The ODs were converted to cell dry weight (CDW) from a standard curve (CDW vs OD) to quantify the free biomass (composed of that from the kefir grains and that from the grape juice) into the culture medium [19].

Concentrations of glucose, fructose, ethanol, glycerol, lactic acid and acetic acid were quantified using a high-performance liquid chromatography (HPLC) 1200 system (Agilent, Waldbronn, Germany) equipped with an ION-300 Organic Acids column (length 300 mm, internal diameter 7.8 mm) with a precolumn IONGUARD™ (polymeric guard column), both obtained from Tecknokroma S. Coop. C. Ltd.a, Barcelona, Spain. Sugars and fermentation products were separated at 60–65 °C using a 0.012 N sulfuric acid aqueous mobile phase flowing at 0.4 mL/min and detected using a refractometer with a refractive index detector (Agilent, model 1200, Santa Clara, CA, USA). Solutions of glucose, fructose, ethanol, glycerol, lactic acid and acetic acid at concentrations between 0.1 and 10.0 g/L were used as standards [20]. Before HPLC analysis, all samples and standards were filtered using a syringe filter (0.22-μm pore size, 25-mm diameter disk filters, Membrane Solutions, Dallas, TX, USA) [20]. All analytical determinations were performed in triplicate.

### 2.5. Antibacterial Activity Quantification

The antibacterial activity against *C. piscicola* CECT 4020 (indicator strain) was quantified using a photometric bioassay and expressed as activity units (AU) per milliliter cell-free supernatant, as described before [19].

### 2.6. Chemical Standards and Reagents

Sodium sulphate anhydrous (99%) was purchased from Merck (Darmstadt, Germany). Ethanol, diethyl ether and hexane, of analytical grade, were purchased from Merck (Darmstadt, Germany). All chemical standards used for identification and the internal standard (4-methyl-2-pentanol) were supplied by Aldrich (Sigma-Aldrich Chemie GmbH, Buchs, Switzerland).

### 2.7. Volatile Compounds Analysis

Liquid–liquid extraction was applied for the isolation of volatile compounds. Specifically, 1 mL of an internal standard solution (1.058 g of 4-methyl-2-pentanol per L of ethanol) was added to a 5-mL sample. Each sample was extracted with 2 mL of diethyl ether-hexane (1:1, *v*/*v*) stirred at 300 rpm for 1 min. After 5 min at cold temperature, the organic extract was dehydrated using anhydrous sodium sulphate. Then, 2 µL of the organic extract was injected into the chromatograph in splitless mode (30 s).

### 2.8. Chromatographic Analyses

The separation, identification and quantification of the volatile compounds were performed on a GC 7820 A gas chromatograph (Agilent Technologies, Santa Clara, CA, USA) coupled with a 5975 Series MSD Agilent mass spectrometer detector. The GC-MS system was equipped with a ZB-Wax column (Phenomenex; 60 m × 0.25 mm × 0.25 µm film thickness). The temperature of the column began at 45 °C, was held for 2 min and increased 2 °C/min to 225 °C. The constant column flow was 1.2 mL/min, using hydrogen as the carrier gas, and the injection port was at 250 °C. The mass spectra were scanned at 70 eV over a mass range from *m*/*z* 10 to 1000.

### 2.9. Identification and Quantification

All volatile compounds were identified using mass spectra (authentic chemicals and Willey spectral library collection), retention indices (RI) and retention times (RT). In some cases, pure reference compounds were used to confirm the results. Identification was considered tentative when based entirely on mass spectral data.

The quantification procedure was conducted using the internal standard quantification method and 4-methyl-2-pentanol as the internal standard. The relative peak areas were calculated in relation to the peak area of the internal standard. Volatile compound determinations were performed in triplicate for each fermented beverage.

### 2.10. Determination of Odor Activity Values

The odor activity values (OAV) were calculated as the ratio between the concentration of a volatile compound in the beverage and its corresponding odor detection threshold (ODT, mg/L) [26]. Volatile compounds with an OAV ≥ 1 were considered to have a direct impact on the aroma of beverages [26]. The ODTs used in this study were determined in water [27,28,29,30].

### 2.11. Enumeration of Microorganisms on the Kefir Grains

To determine the microbial counts in the kefir grains before and after each subculture, four different duplicate cultures were carried out. In the 1st culture, 1 g of kefir grains was inoculated into 20 mL of red table juice (initial pH of 3.99 or 5.99) and incubated for 24 h under the same conditions as those used in the production experiments. The 2nd, 3rd and 4th duplicated cultures were composed of 2, 3 and 4 24-h fed-batch subcultures, respectively.

After the separation of the fermented media by filtration at the end of each culture (24, 48, 72 and 96 h in the 1st, 2nd, 3rd and 4th cultures, respectively), the grains were washed and dried as indicated above. Subsequently, a 0.5 g sample of kefir grains was introduced into a stomacher plastic bag, and subsequently, 50 mL saline (0.8% (*w*/*v*) NaCl) was added. The contents were shredded for 15 min at high speed in a stomacher (Masticator, IUL Instruments, Barcelona, Spain). Then, 2 0.5 mL aliquots of the resulting kefir grain suspension were used to prepare appropriate decimal dilutions in sterile saline solution. These decimal dilutions diluted were plated in triplicate on MRS-A, Carr-A and YEG-C agar media for the enumeration of LAB, AAB and yeasts, respectively. The results were expressed as log CFU (colony forming units) per g of wet kefir grains.

### 2.12. Statistical Analyses

The mean concentrations of the fermentation variables (culture pH, free biomass, microbial counts, glucose, fructose, ethanol, glycerol, lactic acid and acetic acid) and volatile compounds of the different fermented juices were statistically compared using one-way independent analysis of variance (ANOVA) with the Tukey (in case of equal variances) or Games–Howell (in case of unequal variances) post hoc tests after analyzing the homogeneity of the variances with Levene’s test.

All comparisons were made using the software package IBM SPSS Statistics for Windows (Version 21.0, IBM SPSS Inc., Armonk, NY, 2012) with a level of significance of 5%.

Principal component analysis module of the same software package was used to study the relationships among the nonfermented and fermented samples according to their chemical, microbiological and volatile compositions after using Bartlett’s sphericity test to determine the suitability of the data for principal component analysis [12]. The factors with eigenvalues >1.0, according to the Kaiser criterion, were selected for the principal component analysis.

## 3. Results and Discussion

### 3.1. The Fed-Batch Fermentation of Red Table Grapes Juice (Initial pH 3.99) with Kefir Grains

**Figure 1:**
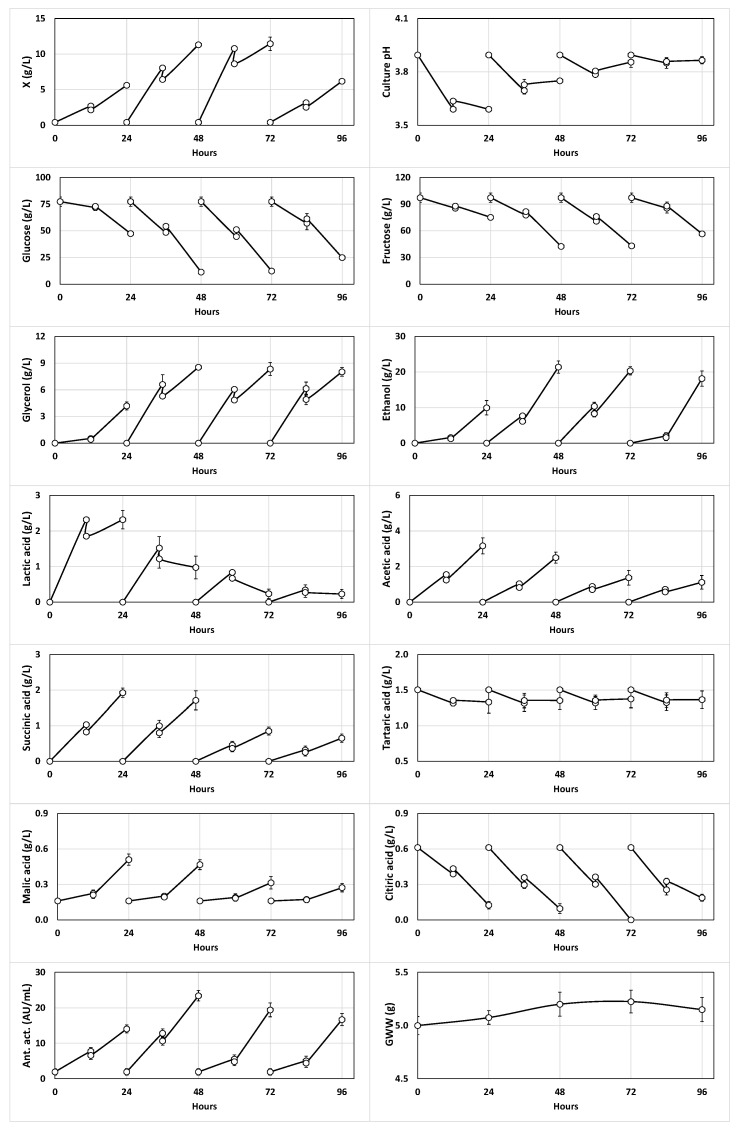
Kinetics of growth (X), culture pH, glucose, fructose, alcohols (glycerol and ethanol) and organic acids (lactic, succinic, tartaric, malic and citric); antibacterial activity (Ant. act.); and grain wet weight (GWW) in the 24-h fed-batch subcultures of red table grape juice at initial pH 3.99 fermented with kefir grains. The different subcultures were fed with fresh juice at 12, 36, 60 and 84 h, respectively.

The fermentation kinetics of red table grape juice with kefir grains in 4 repeated 24-h fed-batch subcultures are shown in Figure 1. From the detailed observation of the culture, it could be noted that the culture pH in every subculture decreased slightly, probably due to the low initial pH (3.99) of the juice and the decreases in the concentrations of organic acids. In fact, the highest concentrations of lactic, acetic, succinic and malic acids (2.32, 3.16, 1.92 and 0.51 g/L, respectively) were obtained in the first subculture, suggesting that the microbial cells from kefir grains progressively lost their ability to acidify the culture medium with the increase in the number of subcultures.

This gradual decrease in the concentrations of organic acids did not seem to be related to a reduction in the counts of lactic acid bacteria (LAB), acetic acid bacteria (AAB) or yeasts because this only occurred in the four subcultures: IV-3.99 (Table 1).

Thus, it seems more appropriate to suppose that the reductions in the concentrations of organic acids were due to their consumption by LAB (e.g., *L. lactis* or *Lactobacillus* sp.) or non-lactose-consuming yeasts (e.g., *Torulaspora delbrueckii* and *Saccharomyces cerevisiae*) present in the kefir grains [31]. Indeed, other researchers have observed that some strains of *L. buchneri* and *L. parabuchneri* are capable of degrading lactic acid to produce 0.5 mole of acetic acid, 0.5 mole of propane-1,2-diol and traces of ethanol [32]. In fermentations developed with mixed cultures of *L. kefiranofaciens* and *S. cerevisiae*, it was observed that lactic acid produced by the lactic acid bacterium was assimilated by yeast [31,33,34]. The results obtained by Felipe et al. [35] showed that some species of yeasts of kefir grains (e.g., *Candida guilliermondii*) can jointly assimilate acetic acid (at concentrations lower than 3.0 g/L) and xylitol from the culture medium. It has also been reported that *S. cerevisiae* strains can use acetic acid and lactic acid [36] or citric acid as carbon sources and that non-Saccharomyces yeasts (e.g., *Candida* sp.) can consume malic acid [37]. Both malic and citric acids can also be metabolized by LAB [37]. However, the concentration of tartaric acid slowly decreases, probably due to its low consumption by LAB because yeasts lack the biochemical pathway for the degradation of this acid [37].

This consumption of organic acids produced a gradual reduction in their concentrations in the fermentation medium, and consequently, the pH drop rate decreased progressively in the different subcultures (Figure 1).

Additionally, the concentrations of free biomass (those released from the kefir grains and the autochthonous biomass of the juice) obtained in the first 24-h subculture (5.62 ± 0.07 g/L) increased in the second and third subcultures to, respectively, 11.31 ± 0.26 (48 h) and 11.45 ± 0.95 g/L (72 h) but decreased to 6.19 ± 0.04 g/L in the last subculture. The increase in free biomass production in subcultures II and III (*p* < 0.05) paralleled the increases in the sugar (glucose and fructose) consumption in these two fermentation cycles (Figure 1). This observation suggests that in subcultures II and III, an activation in the metabolic activity of the free biomass of the juice was produced and the microorganisms from the kefir grains were more adapted to the composition and low pH (3.99) of red grape juice, which was different from the whole milk, the substrate used for kefir grain activation. The observed decrease in free biomass production in subculture IV (72–96 h) could be due to a reduction in the metabolic activity of both the microbial population of the kefir grains and autochthonous biomass of the juice (Figure 1). The latter hypothesis is because, in subculture IV, the microbial population lost its capacity to decrease the culture pH, and the consumption of sugars (glucose and fructose) and synthesis of fermentation products (ethanol, glycerol and organic acids) slowed down (Figure 1).

The production of ethanol and glycerol, probably produced by the yeasts of the kefir grains [38,39], increased until the third subculture and decreased slightly in the fourth (Figure 1), in parallel with the above-mentioned reduction in the metabolic activity of the biomass of the juice and the kefir grains. However, the antibacterial activity, which is due to the production of bacteriocins by LAB [19] and other antimicrobial products (organic acids and alcohols), increased until the 2nd subculture (23.4 AU/mL) but decreased in the following subcultures to 19.4 and 16.7 AU/mL.

Interestingly, at the end of the four subcultures, both the LAB and yeasts counts were higher (*p* < 0.05) than the AAB counts (Table 1), probably due to a better adaptation of the first two microbial groups to the acidity of the juice of red grapes (pH = 3.99). Although it has been indicated that low pH can inhibit the production of organic acids by the LAB and AAB in kefir grains [40], other researchers have observed that some species of Lactobacillus [41] and Lactococcus [42] can grow at pH lower than 3.99. In the case of yeasts, it has been reported that the optimum pH for biomass production by Zygosaccharomyces rouxii DSM 70540 was between 3.50 and 5.00 [24], while for *S. cerevisiae* T73, *S. kudriavzevii* W27 and the hybrid interspecific strain of them, *S. kudriavzevii* IFO 1802T, the optimums were, respectively, 4.76, 3.80 and 4.76 [25]. All these observations indicate that the strains present in the kefir grains can grow at the low pH of the red table grape juice.

The wet weight of the kefir grains increased significantly (*p* < 0.05) from 5.00 g (mass used as inoculum) up to 5.15, 5.40 and 5.40 g (after 24, 48 and 72 h of incubation, respectively), but decreased slightly (although not significantly, *p* > 0.05) to 5.30 g in the fourth subculture compared with the third subculture (Figure 1). The increase in the grain weight has been related to the increase in the number of cells that remain anchored to the grain during fermentation and the increase in the grain matrix weight due to kefiran production [43,44,45,46].

To determine whether the increase in grain weight could be related to the increase in the number of cells that remain anchored to the grain during fermentation, counts of viable LAB, AAB and yeasts in the kefir grains were measured after each subculture.

The results obtained (Table 2) showed that LAB and AAB counts decreased (although not significantly, *p* > 0.05) with the increase in the number of subcultures compared with their corresponding initial counts in the kefir grains. In contrast, the counts of viable yeasts increased (although not significantly, *p* > 0.05), suggesting that this increase could contribute to the increase in grain weight. Moreover, the counts of LAB and AAB decreased slightly at the end of the fourth subculture in parallel with the nonsignificant (*p* > 0.05) decrease in grain weight.

On the other hand, the increase in the wet grain weight in the first three subcultures could also be related to kefiran production from the sugars (glucose and fructose) present in the grape juice. This hypothesis is based on the results obtained by other researchers, who observed that some microorganisms present in kefir grains, such as *L. kefiranofaciens* [44,45] *L. lactis* and *L. mesenteroides* [44], produced kefiran in glucose- [44] or sucrose-containing culture media [45,46].

Regarding the fermentation yields (Table 3), it can be highlighted that yield *Y_X/TSc_* remained approximately constant in the first three subcultures because the free biomass production increased in parallel with increases in the consumption of glucose and fructose. However, *Y_X/TSc_* decreased in the fourth incubation because the decrease in free biomass production was more pronounced than the decreases in the consumption of the two carbon sources (Figure 1).

Although *Y_LA/TSc_*, *Y_AA/TSc_*, *Y_SA/TSc_* and *Y_MA/TSc_* exhibited decreasing profiles (Table 3), the calculated values may not be real due to the hypothesis relating the metabolite production to its possible consumption by some microorganisms present in the kefir grains. However, *Y_Et/TSc_* and *Y_Gly/TSc_* also showed decreasing profiles until the third subculture, although both yields in the fourth incubation were slightly higher than their corresponding values in the first subculture.

### 3.2. Fed-Batch Fermentation of Red Table Grapes Juice (Initial pH 5.99) with Kefir Grains

The low initial pH of red table grape juice (3.99 ± 0.01) seemed to influence the kinetics of the different subcultures, probably because of the different optimum pHs for the growth of each microbial population. According to the results obtained by other researchers, acidic pH favored the growth of yeasts [24,25]. In contrast, the optimum pH range for higher nutrient assimilation by *Streptococcus lactis* and *S. cremoris* strains in a synthetic medium was between 6.00 and 6.50 [21]; for L. casei CECT 4043, it was between 6.50 and 7.00 in deproteinized whey [22]. Moreover, for *Acetobacter* sp. CCTCC M209061, the optimum pH range for biomass production was between 5.0 and 6.0 [23].

**Figure 2:**
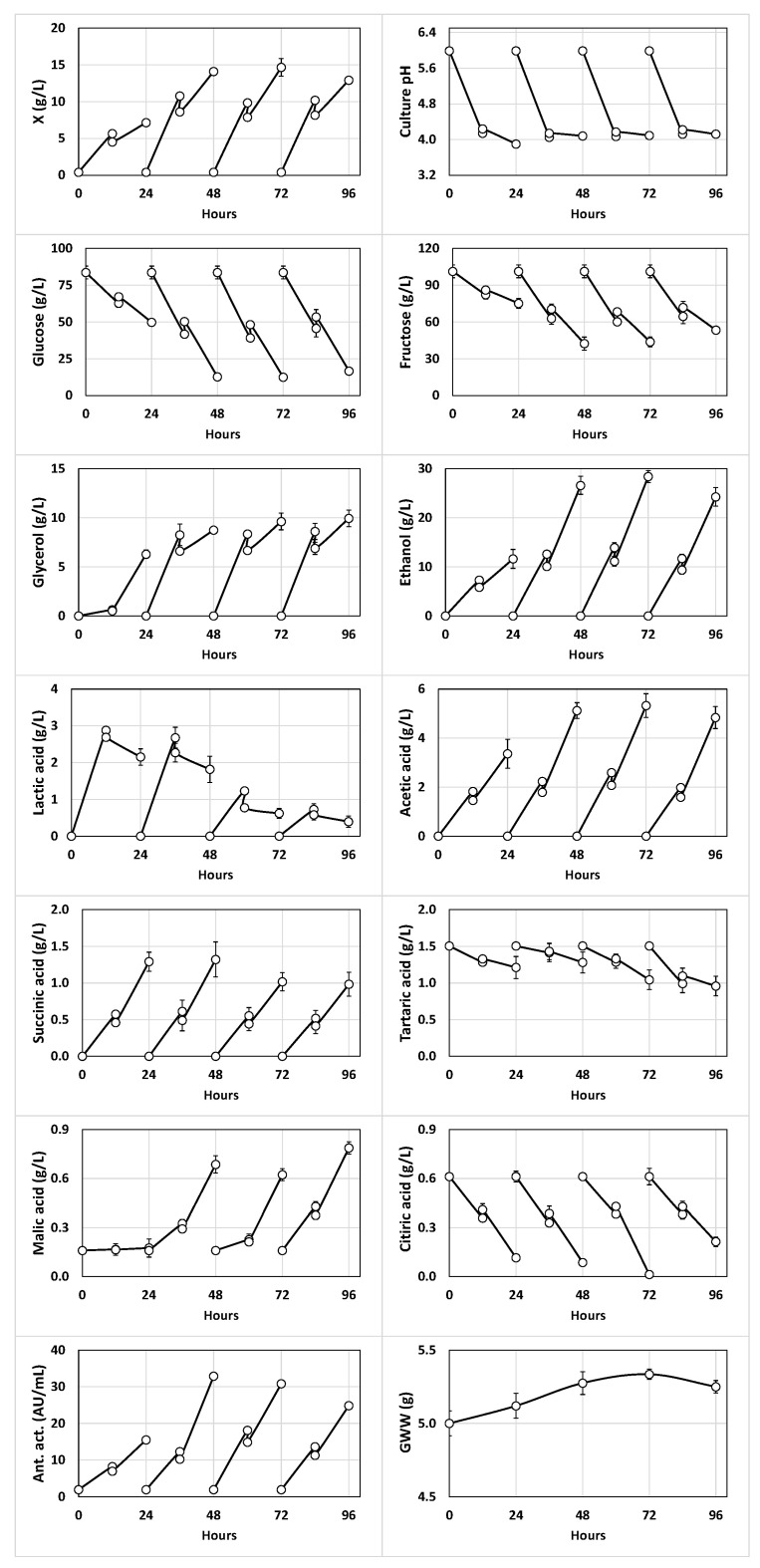
Kinetics of growth (X), culture pH, glucose, fructose, alcohols (glycerol and ethanol) and organic acids (lactic, succinic, tartaric, malic and citric); antibacterial activity (Ant. act.); and grain wet weight (GWW) in the 24-h fed-batch subcultures of red table grape juice at initial pH 5.99 fermented with kefir grains. The different subcultures were fed with fresh juice at 12, 36, 60 and 84 h, respectively.

Considering that kefir grain contains different species of *Lactococcus*, *Lactobacillus* and *Acetobacter* [3,4], a new fermentation (B) was performed (Figure 2) using red table grape juice adjusted to pH 5.99, a more favorable initial pH for the growth of LAB and AAB populations, to determine how this fact affects the fermentation kinetics and production of volatile compounds.

Fermentation B provided increased concentrations (*p* < 0.05) of free biomass, ethanol and glycerol and the higher consumption of total sugars in every fed-batch subculture compared with fermentation A. However, only yields *Y_X/TSc_* and *Y_Et/TSc_* in the different subcultures in fermentation B were always higher than in fermentation A (Table 3 and Table 4).

As observed in fermentation A, *Enterobacteriaciae* and *Pseudomonas* counts in both the kefir-like beverages were considerably low (Table 1). The counts of rod- and coccus-shaped LAB, total mesophilic bacteria and AAB at the end of the four subcultures in fermentation B were higher (*p* < 0.05) than those obtained in the corresponding subcultures in fermentation A (Table 1). However, the counts of yeasts in the four subcultures in fermentations A and B did not show significant differences (*p* > 0.05). Therefore, an initial pH of 5.99 favored the growth of LAB and AAB in comparison with an initial pH of 3.99, but this fact did not significantly affect the counts of yeasts in the red table grape juice.

In the second fed-batch fermentation (Figure 2), the evolution of pH conditioned the production of free biomass, lactic acid and glycerol, which exhibited greater increases in the first 12 h of incubation in the different subcultures when the culture pHs dropped from 5.99 to 4.14 (subculture 1), 4.05 (subculture 2), 4.07 (subculture 3) and 4.12 (subculture 4) just before feeding with fresh grape juice. Although this feeding produced slight increases in the culture pH (up to 4.24, 4.14, 4.17 and 4.22 in subcultures 1, 2, 3 and 4, respectively), slight decreases in this variable were observed in the following 12 h of fermentation in every subculture (Figure 2). Thus, the final culture pH observed in every subculture (3.90, 4.08, 4.09 and 4.12, respectively) was close to the initial pH of the red table grape juice (3.99 ± 0.01).

The production rates of ethanol, acetic acid and succinic acid before and after feeding with fresh juice were similar in every subculture, but the production rate of antibacterial activity after feeding was only higher than that before feeding in the second subculture. In contrast, the synthesis rate of malic acid after feeding was higher than that before feeding from the second subculture (Figure 2).

From the comparison of fed-batch cultures A and B, it could be noted that both the microbial growth and production of ethanol and glycerol did not show the abrupt decrease in the fourth subculture (Figure 2) observed in the previous fed-batch culture at pH 3.99 (Figure 1). This observation suggests that an initial pH of 5.99 favored the stabilization of the microbial populations of the kefir grain during at least 4 fed-batch subcultures.

In addition, the counts of rod and coccus-shaped LAB and yeasts obtained in the 4 subcultures were always very near (first and fourth subcultures in fermentation A) or greater (other subcultures) than 10^6^ CFU/mL. In short, it is foreseeable that the fermented drinks obtained could produce beneficial effects for consumers [6].

When the spontaneous fermentations of the juice from red table grapes with the autochthonous microbiota of the fruit was performed, the counts of LAB (from 1.48 × 10^3^ to 2.50 × 10^3^ CFU/mL) and yeasts (from 6.72 × 10^2^ to 2.15 × 10^4^ CFU/mL) after 24 h of fermentation were relatively low. However, the kefir-like beverages from red table grapes contained counts of LAB and yeasts very near or greater than 10^6^ CFU/mL (Table 1), suggesting that most of the microbial counts in the kefir-like beverages were from the kefir grains.

On the other hand, the growth of kefir grains in fed-batch fermentation B was slightly higher (although not significantly, *p* > 0.05) compared with the previous fed-batch culture (Figure 1 and Figure 2).

Another important aspect considered in the production of fermented beverages with fermentative entities containing yeasts is the production of ethanol. Here, yeasts are important in the production of kefir due to the production of ethanol and carbon dioxide, which provides a drink with a distinctive flavor and stimulating and effervescent characteristics [6,9]. The mean concentrations of ethanol in all the beverages obtained in the different subcultures of fermentations A and B (Figure 1 and Figure 2) were higher than the minimum alcoholic strength (1.2%) fixed by the European Council [47] for alcoholic beverages. According to this criterion, all the kefir-like beverages obtained from red table grapes could be considered alcoholic beverages. However, these beverages contained ethanol concentrations considerably lower than a drink obtained by fermentation of apple with kefir grains (12.27%, *v*/*v*) [48] and some wines produced in Spain [49,50,51,52] (Table 5).

From this observation, it seems reasonable to separate the probiotic cells from the fermented medium to obtain two products with different practical applications. On the one hand, probiotic cells could be separated from the beverage, washed, lyophilized and marketed as an additive for fresh beverages such as milk or fruit juices. On the other hand, the cell-free fermented juice could be marketed as an alcoholic beverage with low ethanol content (between 1.3 and 3.6%, *v*/*v*) and concentration of sugars (~10 g glucose/L and 40 g fructose/L) considerably lower than those of the red table juice (80.51 ± 4.38 g glucose/L and 99.28 ± 2.85 g fructose/L).

### 3.3. Volatile Composition of the Different Fermented Samples

The concentrations of volatile compounds in a fermented beverage depend on the fermentative entity and the quality and type of fermentation substrate as well as the fermentation conditions used [53,54], so that, the volatile composition of the kefir-like beverages obtained from red table grapes is highly influenced by the autochthonous microbiota of the fruit juice and that of the kefir grains, composed of LAB, AAB and yeasts.

Table 6 shows the concentrations (mg/L) of the volatile products detected in the different fermented beverages obtained from fermentations A and B. As can be seen, 68 volatile compounds were quantified in both the fruit juice and fermented beverages, including 21 alcohols, 13 esters, 6 aldehydes, 7 organic acids, 7 ketones, 3 furans, 2 ethers, 6 hydrocarbons and 3 volatile compounds included in other compound families.

As observed, the fed-batch fermentation of red table juices led to an increase in the numbers and concentrations of alcohols, aldehydes (in case of beverages from fermentation B) and organic acids. The concentration of esters also increased, but the number of these volatile compounds increased only in the first subcultures of fermentation A and B (Table 6).

Randazzo et al. [12] observed an increase in the concentrations of organic acids, alcohols, esters and ketones after the batch fermentation of juices from apple, grape, kiwifruit, pomegranate, prickly pear and quince with water kefir microorganisms. In contrast, aldehydes decreased in the fermentations of apple, grape, kiwifruit, pomegranate and quince [12] and in the four beverages from fermentation A (Table 6). The latter was probably due to the low pHs of the juices from apple (pH 3.70), grape (pH 3.61), kiwifruit (pH 3.06), pomegranate (pH 3.66), quince (pH 3.19) [12] and red table grape (pH 3.99). However, in prickly pear juice (pH 6.26), the decrease in aldehyde concentration after fermentation was less pronounced (from 310.56 in the nonfermented juice to 297.32 μg/L in the fermented beverage) than in the above-mentioned fruit juices. These results suggest that the initial pH could play an important role in the production of aldehydes during fermentation of fruit juices.

Fermentations A and B of red table grape juice with milk kefir grains decreased the number of ketones, but a significant decrease (*p* < 0.05) in the concentration of ketones was observed only in fermentation B (Table 6). In contrast, ketone content increased after fermentation of grape, kiwifruit, pomegranate, prickly pear and quince with water kefir microorganisms [12]. However, the fermentation of apple juice with the same fermentation entity only produced a slight increase (from 1.57 to 1.94 μg/L) in the concentrations of these volatile compounds [12].

In the present study, the content and numbers of furans, ethers, hydrocarbons and other compounds decreased with fermentations, as occurred with ketones (Table 6).

The concentrations of alcohols, aldehydes, organic acids, esters and ketones in the fermented beverages, mainly those with OAVs > 1 (Table 7), increased the pleasant or unpleasant keynotes of these kefir-like beverages compared with the nonfermented juice.

Specifically, the presence of 2-methyl-1-propanol (with OAV always higher than 1.0) and 4-ethyl-2-methoxyphenol (Table 6) could confer unpleasant aromas (solvent, bitter and fusel or smoky and gammon-like, respectively) to the fermented beverages. However, the presence of 2-methyl-1-propanol at concentrations much higher than 0.55 mg/L was also detected in four commercialized Galician high-quality orujo spirits [55]: Albariño (210 ± 0.83 mg/L), Mencia (265 ± 1.11 mg/L), Godello (195 ± 3.02) and Treixadura (302 ± 0.78 mg/L).

3-Methyl-1-pentanol with OAV > 1.0 in the kefir-like beverages obtained in subcultures II, III and IV from fermentations A and B provided these beverages with pleasant vinous, herbaceous and cacao notes [56].

The aroma of the kefir-like beverages was negatively affected when the concentration of 3-methyl-1-butanol surpassed its odor detection threshold, since this amyl alcohol is related to alcohol, malt, burned, harsh, nail polish notes [57]. Considering that low concentrations of isoamyl alcohols are related to alcoholic beverages with a light body [55], it can be assumed that the red table grape beverages from subcultures II, III and IV in fermentations A and B have a better body than those of the corresponding first subcultures (Table 6).

Additionally, the ratios of isoamyl alcohol/2-methyl-1-propanol (between 14.4 and 32.7) and 2-methyl-1-propanol/1-propanol (between 2.1 and 3.5) in the different red table grape beverages were higher than 1.0, suggesting that these products could have good organoleptic characteristics according to this criterion [53].

2-Phenylethanol, detected at concentrations between 9.41 and 51.46 mg/L, contributed to the pleasant aroma of red table grape beverages, introducing sweetish and floral nuances in them [53]. This compound was detected at lower concentrations in kefir-like beverages produced from carrot: 54.34 μg/L, fennel: 54.34 μg/L, melon: 393.97 μg/L, strawberry: 215.72 μg/L, tomato: 255.27 μg/L [11], apple: 117.29 μg/L, grape: 588.26 μg/L, kiwifruit: 2241.74 μg/L, pomegranate: 1002.72 μg/L, prickly pear: 514.19 μg/L and quince: 1438.45 μg/L [12].

The presence of 1-hexanol, with aroma descriptions of “coconut-like”, “harsh” and “pungent”, in alcoholic beverages had a positive impact on the aroma at concentrations ≤ 20 mg/L, but at high levels, this volatile compound negatively affected the aroma of these beverages [53]. The presence of 1-hexanol did not negatively affect the aroma of the red table grape beverages here since the concentration of this compound in them was always lower than 20 mg/L (Table 6). Similarly, the kefir-like beverages obtained by the fermentation of juices from vegetables [11] and Mediterranean fruits [12] with water kefir microorganisms had 1-hexanol concentrations lower than 20 mg/L.

Four esters with OAV > 1.0 (pentyl acetate, ethyl hexanoate, 2-phenylethyl acetate and ethyl octanoate), related to fruity/floral/green aromas [11], were detected in the eight kefir-like beverages (Table 6 and Table 7). Similarly, the concentrations of ethyl hexanoate, 2-phenylethyl acetate and ethyl octanoate increased with the fermentation of carrot, fennel, melon, strawberry, tomato [11], apple, grape, kiwifruit, pomegranate, prickly pear and quince [12] with water kefir grains. The production of these compounds during fermentation, related to the metabolic activity of yeast strains present in the kefir grains, contributes to the pleasant aroma (rose and honey) of the beverages [12].

Two aldehydes (2-ethyl hexanal and (E)-2-nonenal) were detected in all fermented samples, although only the (E)-2-nonenal exhibited a considerably higher OAV in all subcultures due to its low threshold (190 ng/L) (Table 6 and Table 7). The latter compound is responsible for important off-flavors (“sawdust” or “plank”) in beverages [58], producing a fatty, tallow, bean, cucumber and woody-like aroma [59].

Organic acids can play an important role in the aroma of alcoholic beverages contributing to their final sensory quality [60,61]. In the kefir-like red table grape beverages, all organic acids were detected at concentrations lower than their corresponding odor thresholds (Table 7), suggesting that the contributions of these volatile compounds to the final aroma of the beverages was very low. A similar trend was observed in wines obtained from Brazilian exotic tropical fruits (cacao, cupuassu, gabiroba, jaboticaba and umbu) and Portuguese grape (Tinta Negra Mole variety) [60].

2-Methyl-2-hexanone, 3-hexanone and 2,6-dimethyl-4-heptanone were detected in the kefir-like beverages at concentrations higher than their corresponding ODTs (Table 7). Therefore, the three ketones can also contribute to the aroma of beverages with fruity or ethereal (in case of 3-hexanone) notes.

However, the contribution of each independent odorant compound to the aroma of an alcoholic beverage can also be influenced by the contributions of other volatile compounds detected, producing a typical aroma of the beverage [62].

### 3.4. Statistical Analysis of the Microbiological, Chemical and Volatile Compositions of the Fermented Samples

Principal component analysis was conducted to interpret the relationships between the microbiological, chemical and volatile compositions of the different fermented samples and identify the main components that best discriminated between the samples analyzed [12,57]. In the analysis of the microbiological, chemical and volatile compositions of the different kefir-like beverages fermented from red table grape juice, Bartlett’s sphericity test (significance = 0.000 < 0.050) indicated that principal component analysis could be applied to the data [12].

**Figure 3:**
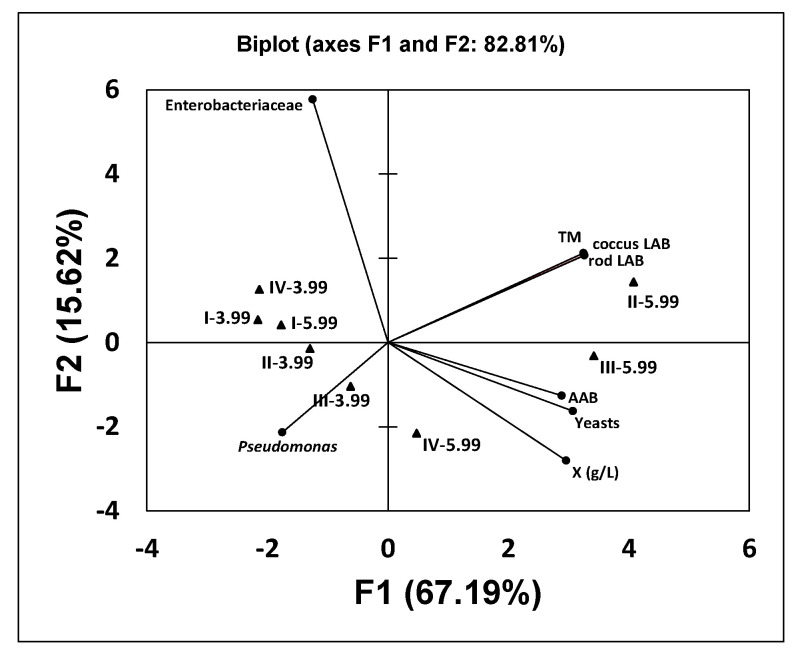
Principal component analysis store plot based on the differences between the microbial counts in the fermented red table grape beverages obtained from fed-batch fermentations A (I−3.99, II−3.99, III−3.99 and IV−3.99) and B (I−5.99, II−5.99, III−5.99 and IV−5.99). TM: counts of total mesophilic bacteria, LAB: lactic acid bacteria, AAB: acetic acid bacteria, X: free biomass concentration.

The principal component analysis of the microbial loads in the different fermented samples with respect to the red table grape juice showed that factor 1 (F1) and 2 (F2), both with eigenvalues higher than 1, accounted for 67.19 and 15.62% of the total variability of the data, respectively, and explained 82.81% of the total variance (Figure 3). According to the biplot graph, Factor 1 was positively correlated with the counts of meshopilic bacteria (in PCA agar), coccus LAB (in M17 agar), rod LAB (in MRS-A agar), AAB (in Carr-A agar) and yeasts (in YEG-C agar) and with the concentrations of free cells (X) in the beverages. That is, according to Factor 1, the beverages obtained in subcultures II-, III- and IV-5.99 (fermentation B) were separated from beverages I-, II-, III- and IV-3.99 (fermentation A) and I-5.99 (fermentation B). This was because beverages II-, III- and IV-5.99 (fermentation B) had higher counts of mesophilic bacteria, coccus and rod LAB, AAB and yeasts and a higher concentration of free biomass (X) than beverages I-, II-, III-, IV-3.99 and I-5.99. These differences could be related to the different initial pHs and nutrient compositions of the table grape juice in fermentations A and B compared with the pasteurized whole milk UHT used for the activation of the kefir grains.

Factor 2 provided a lesser contribution to distinguishing the kefir-like beverages since according to this factor, the samples were closely located along the *x*-axis (Figure 3).

**Figure 4:**
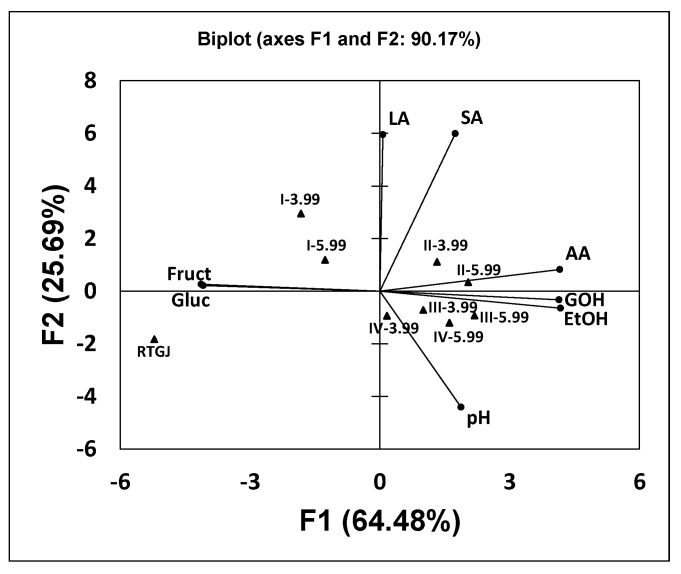
Principal component analysis based on the final pHs and the concentrations of glucose (Gluc), fructose (Fruct), alcohols (EtOH: ethanol and GOH: glycerol) and organic acids (LA: lactic acid, AA: acetic acid, SA: succinic acid) in the nonfermented red table grape juice (RGTJ) and fermented samples obtained from fed-batch fermentations A (I−3.99, II−3.99, III−3.99 and IV−3.99) and B (I−5.99, II−5.99, III−5.99 and IV−5.99).

Principal component analysis based on the chemical compositions of the different beverages (Figure 4) clearly shows the differences between the nonfermented juice and the kefir-like beverages obtained from fermentations A and B. In this case, Factors 1 and 2 accounted for 64.48 and 25.69% of the total variance, respectively. Both factors explained 90.17% of the total variance. The first factor was positively correlated mainly with alcohols (ethanol and glycerol) and acetic acid content and negatively correlated with sugar (glucose and fructose) concentration. According to Factor 1, samples with the highest glucose and fructose concentrations and lowest alcohols and acetic acid levels (the nonfermented juice and kefir-like beverages I-3.99 and I-5.99) were clearly separated from the other fermented samples. Factor 2 separated the samples according to their high lactic and succinic acid concentrations and low final pHs.

In any case, the fermented beverages III-3.99, III-5.99, and IV-5.99 were grouped in the lower-right quarter, indicating that their chemical compositions were similar. Beverage IV-3.99 was also located in this quarter but further away from the group formed by the other three fermented beverages. The same occurred with samples II-3.99 and II-5.99, grouped in the higher-right quarter, and with I-3.99 and I-5.99 grouped in the higher-left quarter. This indicates that the chemical compositions of the fermented beverages were different from those of the nonfermented juice (Figure 4).

**Figure 5:**
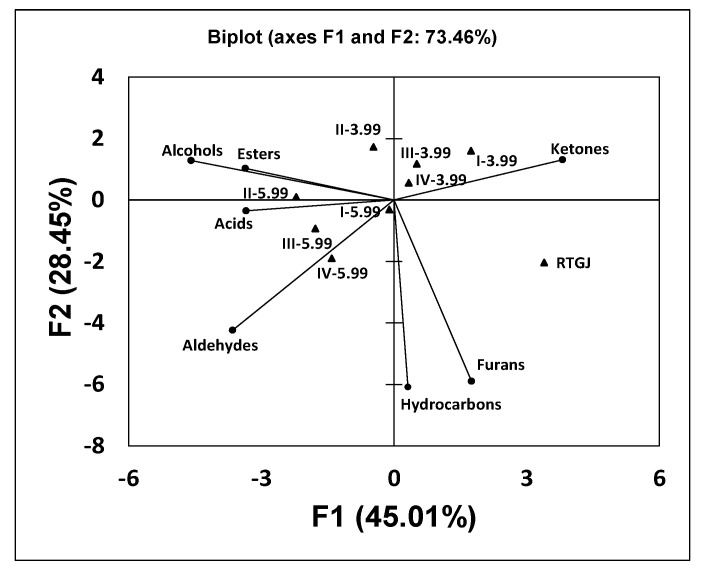
Principal component analysis of volatile compound families (Table 6) in the nonfermented red table grape juice (RGTJ) and fermented samples obtained from fed-batch fermentations A (I−3.99, II−3.99, III−3.99 and IV−3.99) and B (I−5.99, II−5.99, III−5.99 and IV−5.99).

Regarding the volatile compound compositions, principal component analysis was conducted (Figure 5) considering the total concentrations of the main different compound families (alcohols, esters, aldehydes, organic acids, ketones, furans and hydrocarbons) present in the nonfermented red table grape juice and beverages obtained from subcultures I, II, III and IV in fed-batch fermentations A and B (Table 6). Here, 3 factors were obtained with eigenvalues > 1.00, accounting individually for 45.01, 28.45 and 15.34% of the total variance and combined explaining 88.80% of the total variance.

Considering the biplot shown in Figure 5, it can be observed that the nonfermented juice was again the most different sample, being located on the lower-right quarter as an independent sample due to the higher contents of hydrocarbons, furans and ketones (Table 6). Three fermented beverages corresponding to fermentation B (initial pH 5.99) were grouped together in the lower-left quarter (samples I-, III- and IV-5.99) or closely located along the *x*-axis (sample II-5.99) in correspondence with their higher contents in alcohols, esters, aldehydes and organic acids, compared with the nonfermented RTGJ and the corresponding kefir-like beverages from fermentation A. Finally, the four fermented samples corresponding to fermentation A (initial pH 3.99) were grouped in the higher-left (sample II-3.99) and higher-right (samples I-, III- and IV-3.99) quarters in correspondence with their higher ketone contents compared with the corresponding samples from fermentation B (Table 6). This indicates that the initial pH of red grape table juice undoubtedly influenced the aromatic characteristics of the beverages obtained.

Sample I-3.99 was the most different fermented red grape table juice (Figure 5), probably due to the different initial pH (3.99) and media composition between the red table juice and the activation medium (UHT whole milk, pH = 6.75), as indicated before. In the subsequent subcultures (II-, III- and IV-3.99), the concentrations of volatile compounds increased, suggesting that the free biomass and the microorganisms of the kefir grains were more adapted to the acidic pH of the red grape table juice.

In contrast, higher concentrations of volatile compounds were obtained in the four subcultures of fermentation B than in the corresponding subcultures in fermentation A. This observation suggests that in the first subculture (0–24 h) of fermentation B, the microorganisms of the kefir grains adapted better to the red grape table juice since the initial pH (5.99) of this substrate was closer to that of the UHT whole milk.

These results obtained in the principal component analysis (Figure 3, Figure 4 and Figure 5) are in good agreement with the results obtained in the fermentation kinetics of red grape table juices in fermentations A and B (Figure 1 and Figure 2).

## 4. Conclusions

Considering the increasing consumer demand for functional foods, a reproducible fermentation process was designed in this work to produce kefir-like beverages (at initial pH of both 3.99 and 5.99) from red table grape fruits using milk kefir grains, with their distinctive aromatic characteristics.

The results obtained in this study showed the feasibility of producing potentially probiotic beverages with counts of rod- and coccus-shaped LAB and yeasts very near or greater than 10^6^ CFU/mL and good hygienic conditions due to their considerably low *Enterobacteriaceae* and *Pseudomonas* counts. This approach has the additional advantage of the inclusion of table grapes in the production process, allowing for the valorization of the fruit and stimulating its growing and harvesting. The further commercialization of the fermented beverages from red table grapes could increase Spanish producers’ incomes.

## Figures and Tables

**Table 1:** Microbial Counts (Log CFU/mL) in the Red Table Grape Juice (RTGJ) and Fermented Beverages Obtained from the Four Subcultures in Fed-batch Fermentations A (I−3.99, II−3.99, III−3.99 and IV−3.99) and B (I−5.99, II−5.99, III−5.99 and IV−5.99). Each Result Is Shown as Mean ± Standard Deviation of Duplicate Samples and Three Analytical Replications Each.

Beverage	Rod LAB	Mesophilic Coccus LAB	Total Mesophilic	Enterobacteriaceae	*Pseudomonas*	AAB	Yeasts
RTGJ	2.2 ± 0.1 ^a^	3.2 ± 0.1 ^a^	3.3 ± 0.2 ^a^	0.7 ± 0.1 ^a^	0.5 ± 0.1 ^a^	2.0 ± 0.3 ^a^	2.3 ± 0.3 ^a^
I-3.99	6.0 ± 0.4 ^b^	6.0 ± 0.3 ^b^	6.0 ± 0.7 ^b^	0.3 ± 0.1 ^b^	0.3 ± 0.1 ^b^	5.8 ± 0.1 ^b^	7.9 ± 0.1 ^b^
II-3.99	6.5 ± 0.3 ^c^	6.5 ± 0.3 ^c^	6.5 ± 0.5 ^c^	0.3 ± 0.1 ^b^	0.3 ± 0.1 ^b^	6.0 ± 0.5 ^c,b^	8.0 ± 0.7 ^c,b^
III-3.99	6.6 ± 0.3 ^d,c^	6.7 ± 0.2 ^d,c^	6.6 ± 0.4 ^d,c^	nd	nd	5.9 ± 0.3 ^d,b,c^	7.9 ± 0.5 ^d,b,c^
IV-3.99	5.6 ± 0.2 ^e^	5.7 ± 0.3 ^e,b^	5.7 ± 0.1 ^e^	0.3 ± 0.1 ^b^	nd	5.0 ± 0.4 ^e^	6.0 ± 0.6 ^e^
I-5.99	6.9 ± 0.4 ^f^	6.9 ± 0.3 ^f,c,d^	6.9 ± 0.7 ^f,c^	0.3 ± 0.1 ^b^	0.3 ± 0.1 ^b^	6.9 ± 0.7 ^f^	7.6 ± 0.9 ^f,b,c,d^
II-5.99	8.3 ± 0.5 ^g^	8.4 ± 0.4 ^g^	8.4 ± 0.2 ^g^	0.3 ± 0.1 ^b^	nd	7.3 ± 0.5 ^g^	8.3 ± 0.2 ^g,c,d,f^
III-5.99	8.2 ± 0.4 ^h,g^	8.3 ± 0.3 ^h,g^	8.3 ± 0.3 ^h,g^	nd	nd	7.2 ± 0.3 ^h,f,g^	8.2 ± 0.5 ^h,b,c,d,f,g^
IV-6.99	7.3 ± 0.3 ^i,f^	7.3 ± 0.5 ^i,f^	7.2 ± 0.3 ^i,f^	nd	0.3 ± 0.1 ^b^	7.1 ± 0.6 ^i,f,g,h^	8.2 ± 0.3 ^i,c,d,f,g,h^

Means within columns followed by the same letter are not significantly different by Tukey or Games-Howell post hoc tests (*p* = 0.05) after a significant ANOVA (*p* < 0.05).

**Table 2:** Counts of Lactic Acid Bacteria (LAB), Acetic Acid Bacteria (AAB) and Yeasts in the Kefir Grains before Inoculation (t = 0 h) and after 24, 48, 72 and 96 h. Results Are Shown as Means ± Standard Deviations of Two Experiments and Three Analytical Replications Each.

	Before Inoculation	At 24 h	At 48 h	At 72 h	At 96 h
LAB (CFU/g)	9.9 ± 1.3 × 10^7^	9.6 ± 1.5 × 10^7^	9.5 ± 1.4 × 10^7^	9.2 ± 1.7 × 10^7^	9.0 ±1.4 × 10^7^
AAB (CFU/g)	6.8 ± 1.0 × 10^6^	6.6 ± 1.2 × 10^6^	6.5 ±1.2 × 10^6^	6.5 ± 1.5 × 10^6^	6.0 ±1.1 × 10^6^
Yeasts (CFU/g)	7.8 ± 1.6 × 10^7^	8.1 ± 1.4 × 10^7^	8.9 ± 1.5 × 10^7^	9.2 ±1.9 × 10^7^	9.4 ±1.6 × 10^7^

**Table 3:** Fermentation Yields Calculated at the End of Every Subculture in Fed-Batch Fermentation A. *Y_X/TSc_*, *Y_LA/TSc_*, *Y_AA/TSc_*, *Y_SA/TSc_*, *Y_MA/TSc_*, *Y_Et/TSc_* and *Y_Gly/TSc_* are respectively, the Mean Yields (g/g) of Free Biomass (X), Lactic Acid (LA), Acetic Acid (AA), Succinic Acid (SA), Malic Acid (MA), Ethanol (Et) and Glycerol (Gly) on Total Sugars Consumed (TSc).

	Subcultures			
Variables	I	II	III	IV
*Y_X/TSc_*	0.100	0.090	0.093	0.062
*Y_LA/TSc_*	0.045	0.008	0.002	0.002
*Y_AA/TSc_*	0.061	0.021	0.012	0.012
*Y_SA/TSc_*	0.037	0.014	0.007	0.007
*Y_MA/TSc_*	0.007	0.003	0.001	0.001
*Y_Et/TSc_*	0.191	0.177	0.171	0.195
*Y_Gly/TSc_*	0.080	0.071	0.070	0.086

**Table 4:** Fermentation Yields Calculated at the End of Every Subculture in Fed-Batch Fermentation B. *Y_X/TSc_*, *Y_LA/TSc_*, *Y_AA/TSc_*, *Y_SA/TSc_*, *Y_MA/TSc_*, *Y_Et/TSc_*, and *Y_Gly/TSc_* are respectively, the Mean Yields (g/g) of Free Biomass (X), Lactic Acid (LA), Acetic Acid (AA), Succinic Acid (SA), Malic Acid (MA), Ethanol (Et) and Glycerol (Gly) on Total Sugars Consumed (TSc).

	Subcultures			
Variables	I	II	III	IV
*Y_X/TSc_*	0.112	0.106	0.111	0.109
*Y_LA/TSc_*	0.036	0.014	0.005	0.003
*Y_AA/TSc_*	0.056	0.039	0.041	0.042
*Y_SA/TSc_*	0.022	0.010	0.008	0.009
*Y_MA/TSc_*	0.000	0.004	0.004	0.005
*Y_Et/TSc_*	0.194	0.205	0.221	0.211
*Y_Gly/TSc_*	0.105	0.067	0.075	0.086

**Table 5:** Comparisons of the Mean Alcoholic Content (%, *v*/*v*) in the Fermented Samples from the Four Subcultures of Fed-Batch Fermentations A and B with Other Fermented Beverages. The Soluble Solids Content of the Table Grape Juice was 15.6 ± 0.02 °Brix.

	Subcultures of 24 h				References
	I	II	III	IV	
Fermentation A	1.3	2.7	2.6	2.3	This work
Fermentation B	1.5	3.4	3.6	3.1	This work
Apple kefir	12.27, after 120 h of fermentation				Viana et al. [48]
Wines					
Spanish barrel-aged red wines	12.0–14.5				Garde et al. [49]
Rojal (red wine)	12.52				Sánchez-Palomo et al. [50]
Moravia Dulce (red wine)	12.70				
Tortosí (red wine)	12.72				
Cabernet-sauvignon (white wine)	11.8				Vilanova et al. [51]
Pinot noir (white wine)	13.5				
Tempranillo (white wine)	11.6				
Merlot (white wine)	13.1				
Chardonnay (red wine)	14.1				
Pinot blanc (red wine)	11.6				
Pinot gris (red wine)	14.6				
Riesling (red wine)	11.0				
Sauvignon blanc (red wine)	13.6				
Gewürztraminer (red wine)	14.3				
Albariño (white wine)	13.82				Vilanova and Freire [52]
Loureiro (white wine)	12.75				

**Table 6:** Mean Concentrations (mg/L) and Standard Deviations of the Volatile Organic Compounds Detected in the Nonfermented Red Table Grape Juice (RTGJ) and in Different Kefir-like Beverages Obtained from the Four Subcultures in Fed-batch Fermentation A (I−3.99, II−3.99, III−3.99 and IV−3.99) and B (I−5.99, II−5.99, III−5.99, and IV−5.99). Results Are Shown as Means ± Standard Deviations of Two Experiments and Three Analytical Replications Each.

			Fermentation A				Fermentation B			
nº	Compound	RTGJ	I−3.99	II−3.99	III−3.99	IV−3.99	I−5.99	II−5.99	III−5.99	IV−5.99
	**Alcohols**									
1	1-Propanol	N.d.	N.d.	1.87 ± 1.29	1.19 ± 0.51	1.21 ± 0.00	0.61 ± 0.00	2.84 ± 1.37	1.36 ± 0.13	1.75 ± 0.19
2	2-Methyl-1-propanol	N.d.	1.65 ± 0.00	4.29 ± 0.70	2.88 ± 0.43	3.16 ± 0.00	1.93 ± 0.05	5.95 ± 0.00	4.71 ± 0.91	5.84 ± 0.11
3	3-Methyl-1-pentanol	N.d.	N.d.	0.17 ± 0.01	0.19 ± 0.06	0.14 ± 0.00	N.d.	0.23 ± 0.01	0.13 ± 0.02	0.13 ± 0.03
4	3-Methyl-1-butanol	N.d.	43.21 ± 0.00	119.24 ± 1.03	94.07 ± 1.77	62.86 ± 0.00	27.78 ± 1.54	125.36 ± 8.62	107.13 ± 1.34	92.96 ± 2.09
5	2-Ethyl-2-hexen-1-ol	N.d.	0.65 ± 0.00	N.d.	N.d.	N.d.	0.87 ± 0.00	N.d.	N.d.	N.d.
6	2-Phenylethanol	N.d.	9.41 ± 0.00	33.33 ± 1.34	20.34 ± 3.93	24.96 ± 0.00	45.90 ± 0.03	51.46 ± 10.61	51.16 ± 7.96	44.12 ± 3.10
7	4-Ethyl-2-methoxyphenol	N.d.	N.d.	0.10 ± 0.07	0.09 ± 0.038	0.12 ± 0.00	0.30 ± 0.00	0.15 ± 0.08	0.13 ± 0.00	0.13 ± 0.00
8	3-Methyl-4-heptanol	N.d.	0.10 ± 0.00	0.13 ± 0.04	0.11 ± 0.06	0.14 ± 0.00	0.17 ± 0.04	0.21 ± 0.02	0.24 ± 0.16	0.25 ± 0.01
9	1-Hexanol	N.d.	4.03 ± 0.00	3.47 ± 0.40	3.26 ± 0.27	2.99 ± 0.00	3.23 ± 0.24	2.59 ± 0.35	2.91 ± 0.00	2.63 ± 0.00
10	3-Methyl-4-penten-1-ol	N.d.	0.11 ± 0.00	N.d.	N.d.	N.d.	N.d.	N.d.	N.d.	N.d.
11	1-Octin-3-ol	N.d.	0.04 ± 0.00	N.d.	N.d.	N.d.	N.d.	N.d.	N.d.	N.d.
12	Cis-3-methylcyclohexanol	N.d.	0.13 ± 0.00	0.14 ± 0.00	0.14 ± 0.01	0.11 ± 0.00	0.13 ± 0.00	0.15 ± 0.00	0.18 ± 0.01	0.14 ± 0.05
13	Trans-2-ethyl-2-hexen-1-ol	N.d.	0.21 ± 0.00	0.19 ± 0.02	0.19 ± 0.04	0.15 ± 0.00	0.14 ± 0.03	0.19 ± 0.02	0.22 ± 0.00	0.17 ± 0.00
14	4-Cyclohexene-1,2-diol	N.d.	0.11 ± 0.00	N.d.	N.d.	N.d.	N.d.	N.d.	N.d.	N.d.
15	1,3-Butanediol	N.d.	0.14 ± 0.00	1.36 ± 0.13	1.40 ± 0.81	1.40 ± 0.00	1.28 ± 0.00	2.36 ± 0.24	2.05 ± 0.50	2.02 ± 0.35
16	2-Butyl-1-octanol	N.d.	N.d.	0.06 ± 0.09	0.05 ± 0.08	0.06 ± 0.00	0.09 ± 0.00	0.06 ± 0.00	0.12 ± 0.00	0.14 ± 0.00
17	2-Furanmethanol	0.372 ± 0.001	N.d.	N.d.	N.d.	N.d.	N.d.	N.d.	N.d.	N.d.
18	2-Cyclohexyl-3-isopropyl-pent-4-en-2-ol	0.244 ± 0.020	N.d.	N.d.	N.d.	N.d.	N.d.	N.d.	N.d.	N.d.
19	1-Hexadecanol	0.143 ± 0.011	N.d.	N.d.	N.d.	N.d.	N.d.	N.d.	N.d.	N.d.
20	2-Hexadecanol	0.142 ± 0.007	N.d.	N.d.	N.d.	N.d.	N.d.	N.d.	N.d.	N.d.
21	(Z)-2-Hexen-1-ol	0.066 ± 0.001	N.d.	N.d.	N.d.	N.d.	N.d.	N.d.	N.d.	N.d.
	**Number of COV**	5	12	12	12	12	12	12	12	12
	**Total concentrations**	0.967 ± 0.010 ^a^	59.81 ± 9.50 ^b^	164.37 ± 26.53 ^c^	123.91 ± 20.68 ^d,c^	97.32 ± 14.40 ^e^	82.43 ± 11.34 ^f^	191.54 ± 28.87 ^g,c^	170.36 ± 25.25 ^h,c^	150.28 ± 21.86 ^c^
	**Esters**									
22	Pentyl acetate	N.d.	0.62 ± 0.00	2.56 ± 0.15	1.62 ± 0.11	0.30 ± 0.00	1.27 ± 0.00	1.79 ± 0.26	1.71 ± 0.03	0.87 ± 0.19
23	Ethyl hexanoate	N.d.	0.54 ± 0.00	1.12 ± 0.40	0.81 ± 0.14	0.26 ± 0.00	0.58 ± 0.00	0.61 ± 0.17	1.01 ± 0.07	0.79 ± 0.01
24	2-Methylamyl acetate	N.d.	0.31 ± 0.00	N.d.	N.d.	N.d.	N.d.	N.d.	N.d.	N.d.
25	2-Phenylethyl acetate	N.d.	0.31 ± 0.00	0.46 ± 0.14	0.36 ± 0.19	0.32 ± 0.00	0.81 ± 0.26	0.92 ± 0.32	0.76 ± 0.07	0.63 ± 0.06
26	3-(Methylthio) propylnonanoate	N.d.	3.37 ± 0.00	4.96 ± 1.97	3.26 ± 2.13	7.48 ± 0.01	5.87 ± 0.92	6.12 ± 0.96	3.27 ± 1.30	6.93 ± 0.21
27	2,2-Dimethyl-1-propanol-acetate	N.d.	0.69 ± 0.00	N.d.	N.d.	N.d.	1.69 ± 0.00	1.75 ± 0.29	N.d.	N.d.
28	Ethyl octanoate (ethyl caprylate)	N.d.	0.24 ± 0.00	0.70 ± 0.14	0.50 ± 0.03	0.13 ± 0.00	0.32 ± 0.02	0.34 ± 0.06	0.44 ± 0.05	0.26 ± 0.09
29	2-Oxo-2-phenylethyl-(benzoylsulfanyl) acetate	0.27 ± 0.00	N.d.	N.d.	N.d.	N.d.	0.13 ± 0.00	N.d.	N.d.	N.d.
30	3-Methylene-4-pentenyl acrylate	0.82 ± 0.01	N.d.	N.d.	N.d.	N.d.	N.d.	N.d.	N.d.	N.d.
31	2-Propenyl formate	2.23 ± 0.02	N.d.	N.d.	N.d.	N.d.	N.d.	N.d.	N.d.	N.d.
32	Heptyl 2-(methoxycarbonylamino) propanoate	0.64 ± 0.00	N.d.	N.d.	N.d.	N.d.	N.d.	N.d.	N.d.	N.d.
33	2-O-cyclobutyl 1-O-heptyl oxalate	0.46 ± 0.01	N.d.	N.d.	N.d.	N.d.	N.d.	N.d.	N.d.	N.d.
34	S-heptyl propanethioate	0.64 ± 0.01	N.d.	N.d.	N.d.	N.d.	N.d.	N.d.	N.d.	N.d.
	**Number of COV**	6	7	5	5	5	7	6	5	5
	**Total concentrations**	5.07 ± 0.63 ^a^	6.09 ± 0.91 ^b,a^	9.79 ± 1.46 ^c^	6.56 ± 0.96 ^d,a,b^	8.50 ± 2.05 ^e,a,b,c,d^	10.67 ± 1.61 ^f,c,e^	11.54 ± 1.70 ^g,c,f^	7.19 ± 0.98 ^h,b,c,d,e^	9.48 ± 1.89 ^c,d,e,f,g,h^
	**Aldehydes**									
35	2-Ethyl hexanal	N.d.	N.d.	0.18 ± 0.06	0.15 ± 0.07	0.20 ± 0.00	0.18 ± 0.13	0.30 ± 0.05	0.40 ± 0.21	0.39 ± 0.03
36	3-(Methylthio)-nonanal	N.d.	0.064 ± 0.001	N.d.	N.d.	N.d.	N.d.	N.d.	N.d.	N.d.
37	(E)-2-nonenal	N.d.	0.09 ± 0.00	0.19 ± 0.06	0.18 ± 0.03	0.13 ± 0.01	0.08 ± 0.00	0.12 ± 0.02	0.15 ± 0.01	0.14 ± 0.03
38	2,2-Dimethyl propanal	N.d.	N.d.	0.01 ± 0.00	0.02 ± 0.00	N.d.	0.17 ± 0.00	0.22 ± 0.00	0.22 ± 0.00	0.16 ± 0.00
39	(E)-2-Hexenal	0.29 ± 0.00	N.d.	N.d.	N.d.	N.d.	N.d.	N.d.	N.d.	N.d.
40	5-Methyl-2-furancarboxaldehyde	0.09 ± 0.00	N.d.	N.d.	N.d.	N.d.	N.d.	N.d.	N.d.	N.d.
	**Number of COV**	2	2	3	3	2	3	3	3	3
	**Total concentrations**	0.39 ± 0.12 ^a^	0.15 ± 0.04 ^b^	0.37 ± 0.09 ^c,a^	0.35 ± 0.08 ^d,a,c^	0.33 ± 0.09 ^e,a,b,c,d^	0.43 ± 0.09 ^f,a,c,d,e^	0.65 ± 0.13 ^g,c,f^	0.76 ± 0.16 ^h,g^	0.69 ± 0.15 ^g,h^
	**Organic acids**									
41	Hexanoic acid (caproic)	N.d.	0.85 ± 0.00	1.16 ± 0.69	1.45 ± 1.21	2.59 ± 0.01	4.49 ± 1.76	3.36 ± 2.82	2.01 ± 0.21	1.76 ± 0.56
42	Octanoic acid (caprylic)	N.d.	0.56 ± 0.00	1.22 ± 0.99	1.83 ± 1.63	1.68 ± 0.01	4.83 ± 0.01	3.41 ± 3.28	2.04 ± 0.12	2.35 ± 1.10
43	Nonanoic acid (pelargonic)	N.d.	N.d.	0.09 ± 0.06	0.11 ± 0.08	0.07 ± 0.00	0.16 ± 0.00	0.20 ± 0.11	0.24 ± 0.12	0.25 ± 0.02
44	Acetic acid	0.70 ± 0.03	1.05 ± 0.00	1.04 ± 0.36	1.62 ± 0.53	3.90 ± 0.47	0.95 ± 0.24	0.87 ± 0.03	1.53 ± 0.11	2.08 ± 0.43
45	Pentanoic acid	N.d.	0.08 ± 0.00	0.22 ± 0.17	0.25 ± 0.22	0.31 ± 0.00	0.20 ± 0.11	0.43 ± 0.25	0.43 ± 0.36	0.37 ± 0.21
46	4-Methyl pentanoic acid	0.11 ± 0.00	N.d.	N.d.	N.d.	N.d.	N.d.	N.d.	N.d.	N.d.
47	2-Methyl-hexanoic acid	N.d.	0.16 ± 0.00	0.34 ± 0.06	0.21 ± 0.05	0.16 ± 0.00	0.60 ± 0.00	0.62 ± 0.10	0.63 ± 0.36	0.52 ± 0.25
	**Number of COV**	2	5	6	6	6	6	6	6	6
	**Total concentrations**	0.82 ± 0.26 ^a^	2.69 ± 0.43 ^b^	4.06 ± 0.53 ^c^	5.47 ± 0.81 ^d,c^	8.72 ± 1.53 ^e^	11.23 ± 2.11 ^f^	8.89 ± 1.47 ^g,e^	6.89 ± 0.86 ^h,d,e,g^	7.33 ± 0.98 ^d,e,g,h^
	**Ketones**									
48	3,3-Dimethyl-2-butanone	0.04 ± 0.00	N.d.	N.d.	N.d.	N.d.	N.d.	N.d.	N.d.	N.d.
49	4-Methyl-2-hexanone	0.05 ± 0.00	0.05 ± 0.00	0.05 ± 0.00	0.05 ± 0.00	0.05 ± 0.00	0.04 ± 0.00	0.04 ± 0.00	0.06 ± 0.00	0.05 ± 0.00
50	3-Hexanone	0.16 ± 0.00	0.16 ± 0.00	0.14 ± 0.00	0.16 ± 0.00	0.16 ± 0.00	0.12 ± 0.00	0.14 ± 0.00	0.12 ± 0.00	0.11 ± 0.00
51	2,6-Dimethyl-4-heptanone	0.27 ± 0.00	0.66 ± 0.00	0.67 ± 0.00	0.71 ± 0.00	0.77 ± 0.01	0.47 ± 0.00	0.56 ± 0.00	0.39 ± 0.00	0.52 ± 0.00
52	5-Dodecanone	0.09 ± 0.00	N.d.	N.d.	N.d.	N.d.	N.d.	N.d.	N.d.	N.d.
53	Dihydroxyacetone	0.35 ± 0.00	N.d.	N.d.	N.d.	N.d.	N.d.	N.d.	N.d.	N.d.
54	1-Acetyloxy-2-propanone	0.13 ± 0.00	N.d.	N.d.	N.d.	N.d.	N.d.	N.d.	N.d.	N.d.
	**Number of COV**	7	3	3	3	3	3	3	3	3
	**Total concentrations**	1.09 ± 0.12 ^a^	0.87 ± 0.24 ^b,a^	0.86 ± 0.25 ^c,a,b^	0.93 ± 0.26 ^d,a,b,c^	0.98 ± 0.28 ^e,a,b,c,d^	0.63 ± 0.17 ^f,b^	0.75 ± 0.21 ^g,b,c,d,f^	0.57 ± 0.14 ^h,b,f,g^	0.68 ± 0.19 ^b,d,f,g,h^
	**Furans**									
55	3-Butyldihydro-2(3H)-furanone	0.10 ± 0.00	N.d.	N.d.	N.d.	N.d.	N.d.	N.d.	N.d.	N.d.
56	5-Methyl-2(3H)-furanone	0.96 ± 0.02	N.d.	N.d.	N.d.	N.d.	N.d.	N.d.	N.d.	N.d.
57	Tetrahydro-2,5-dimethyl-furan	0.10 ± 0.00	0.20 ± 0.00	0.18 ± 0.00	0.22 ± 0.00	0.38 ± 0.00	0.72 ± 0.00	0.49 ± 0.00	0.43 ± 0.00	0.64 ± 0.00
	**Number of COV**	3	1	1	1	1	1	1	1	1
	**Total concentrations**	1.16 ± 0.01 ^a^	0.20 ± 0.12 ^b^	0.18 ± 0.11 ^c,b^	0.22 ± 0.12 ^d,b,c^	0.38 ± 0.22 ^e,b,c,d^	0.72 ± 0.42 ^f,a,b,d,e^	0.49 ± 0.28 ^g,b,c,e,f^	0.43 ± 0.25 ^h,b,d,e,g^	0.64 ± 0.37 ^a,b,d,e,f,g^
	**Ethers**									
58	1-Butoxy-3-methyl-2-butene	0.14 ± 0.00	N.d.	N.d.	N.d.	N.d.	N.d.	N.d.	N.d.	N.d.
59	Ethyl-1-propenyl ether	0.19 ± 0.00	N.d.	N.d.	N.d.	N.d.	N.d.	N.d.	N.d.	N.d.
	**Number of COV**	2	N.d.	N.d.	N.d.	N.d.	N.d.	N.d.	N.d.	N.d.
	**Total concentrations**	0.33 ± 0.04	N.d.	N.d.	N.d.	N.d.	N.d.	N.d.	N.d.	N.d.
	**Hydrocarbons**									
60	2,4-Dimethyl-2-pentene	0.06 ± 0.00	N.d.	N.d.	N.d.	N.d.	N.d.	N.d.	N.d.	N.d.
61	(Z)-3-Dodecene	0.26 ± 0.00	0.26 ± 0.00	0.24 ± 0.00	0.23 ± 0.00	0.29 ± 0.00	0.23 ± 0.00	0.22 ± 0.00	0.29 ± 0.00	0.29 ± 0.00
62	4-Cyclopenten-1,3-dione	0.45 ± 0.01	N.d.	N.d.	N.d.	N.d.	N.d.	N.d.	N.d.	N.d.
63	1,2,3-Trimethyl-benzene	0.42 ± 0.01	0.35 ± 0.00	0.33 ± 0.00	0.35 ± 0.00	0.36 ± 0.00	0.41 ± 0.00	0.44 ± 0.00	0.45 ± 0.00	0.53 ± 0.00
64	Butylhydroxytoluene	1.43 ± 0.02	1.53 ± 0.02	1.45 ± 0.01	1.63 ± 0.02	1.78 ± 0.01	1.16 ± 0.02	1.61 ± 0.02	1.83 ± 0.03	1.95 ± 0.02
65	Trichloromethane	0.27 ± 0.01	N.d.	N.d.	N.d.	N.d.	0.32 ± 0.00	N.d.	N.d.	0.32 ± 0.01
	**Number of COV**	6	3	3	3	3	4	3	3	4
	**Total concentrations**	2.90 ± 0.48 ^a^	2.14 ± 0.59 ^b^	2.02 ± 0.56 ^c,a,b^	2.22 ± 0.64 ^d,a,b,c^	2.43 ± 0.69 ^e,a,b,c,d^	2.13 ± 0.43 ^f,a,b,c,d,e^	2.27 ± 0.63 ^a,b,c,d,f^	2.57 ± 0.71 ^a,b,c,d,f^	3.09 ± 0.73 ^a,b,c^
	**Other compounds**									
66	3-Trifluoroacetoxy dodecane	0.08 ± 0.00	N.d.	0.04 ± 0.00	N.d.	N.d.	N.d.	N.d.	N.d.	N.d.
67	1-Hydroperoxyhexane	0.14 ± 0.00	N.d.	N.d.	N.d.	N.d.	N.d.	N.d.	N.d.	N.d.
68	Cis-2-methyl-3-propyl-oxirane	0.34 ± 0.01	N.d.	N.d.	N.d.	N.d.	0.09 ± 0.00	N.d.	N.d.	0.06 ± 0.00
	**Number of COV**	3	0	1	0	0	1	0	0	1
	**Total concentrations**	0.57 ± 0.14 ^a^	0 ^b^	0.04 ± 0.02 ^c^	0 ^b^	0 ^b^	0.09 ± 0.05 ^c^	0 ^b^	0 ^b^	0.06 ± 0.03 ^c^

N.d.: not detected. Means within rows followed by the same letter are not significantly different by Tukey or Games–Howell post hoc tests (*p* = 0.05) after a significant ANOVA (*p* < 0.05).

**Table 7:** Odor Activity Values (OAV) and Odor Detection Thresholds (ODT) of the Volatile Organic Compounds Detected in the Nonfermented Red Table Grape Juice (RTGJ) and in Different Kefir-like Beverages Obtained from the Four Subcultures in Fed-batch Fermentations A (I−3.99, II−3.99, III−3.99 and IV−3.99) and B (I−5.99, II−5.99, III−5.99 and IV−5.99).

				OVA								
No.	Compound	ODT (mg/L)	Descriptor	RTGJ	I−3.99	II−3.99	III−3.99	IV−3.99	I−5.99	II−5.99	III−5.99	IV−5.99
1	1-Propanol	9 [28]	ripe fruit, alcohol [63]			0.21	0.13	0.14	0.07	0.32	0.15	0.20
2	2-Methyl-1-propanol	0.55 [28]	alcohol, banana, medicinal, solvent, nail polish [53]		3.01	7.81	5.23	5.75	3.51	10.82	8.56	10.61
3	3-Methyl-1-pentanol	0.0075 [28]	vinous, herbaceous, cacao [56]			2.33	2.49	1.84		3.03	1.71	1.76
4	3-Methyl-1-butanol	50–70 [28]	whiskey, malt, burned, harsh, nail polish [57]		0.72 *	1.99 *	1.57 *	1.05 *	0.46 *	2.09 *	1.79 *	1.55 *
5	2-Ethyl-2-hexen-1-ol	Nf	citrus, floral, sweet [64]									
6	2-Phenylethanol	0.5642 [28]	rose, sweetish, perfumed [53]		16.67	59.07	36.05	44.24	81.34	91.21	90.68	78.19
7	4-Ethyl-2-methoxyphenol	0.08925 [28]	smoky, gammon-like [65]			1.17	0.99	1.36	3.36	1.68	1.48	1.46
8	3-Methyl-4-heptanol	0.078–0.420 [28]	Nf		0.41 *	0.52 *	0.43 *	0.58 *	0.69 *	0.84 *	0.96 *	1.00 *
9	1-Hexanol	0.0056 [28]	coconut, harsh, pungent [53]		71.96	61.96	58.25	53.46	57.70	46.18	52.00	46.93
10	3-Methyl-4-penten-1-ol	Nf	Nf									
11	1-Octin-3-ol	Nf	Nf									
12	Cis-3-methylcyclohexanol	Nf	Nf									
13	Trans-2-ethyl-2-hexen-1-ol	Nf	Nf									
14	4-Cyclohexene-1,2-diol	Nf	Nf									
15	1,3-Butanediol	10–20 [28]	Woody [66]		0.01 *	0.09 *	0.09 *	0.09 *	0.08 *	0.16 *	0.14 *	0.13 *
16	2-Butyl-1-octanol	Nf	Nf									
17	2-Furanmethanol	4.5 [28]	burned sugar [64]	0.08								
18	2-Cyclohexyl-3-isopropyl-pent-4-en-2-ol	Nf	Nf									
19	1-Hexadecanol	0.75 [30]	floral, waxy [67]	0.19								
20	2-Hexadecanol	Nf	Nf									
21	(Z)-2-Hexen-1-ol	0.3593 [28]	green grass, herb [68]	0.184								
22	Pentyl acetate	0.043 [28]	fruity [69]		14.46	59.60	37.67	7.02	29.49	41.70	39.84	20.19
23	Ethyl hexanoate	0.005 [28]	Fruity [70]		108.00	223.20	162.80	52.20	115.60	122.20	201.80	157.20
24	2-Methylamyl acetate	Nf	Nf									
25	2-Phenylethyl acetate	0.25 [28]	rose, honey [71]		1.24	1.83	1.44	1.30	3.25	3.69	3.02	2.54
26	3-(Methylthio) propylnonanoate	Nf	Nf									
27	2,2-Dimethyl-1-propanol-acetate	Nf	Nf									
28	Ethyl octanoate (ethyl caprylate)	0.0193 [28]	fruity, floral [71]		12.54	36.17	26.11	6.94	16.74	17.67	22.90	13.42
29	2-Oxo-2-phenylethyl-(benzoylsulfanyl)acetate	Nf	Nf									
30	3-Methylene-4-pentenyl acrylate	Nf	Nf									
31	2-Propenyl formate	Nf	Nf									
32	Heptyl 2-(methoxycarbonylamino) propanoate	Nf	Nf									
33	2-O-cyclobutyl 1-O-heptyl oxalate	Nf	Nf									
34	S-heptyl propanethioate	Nf	Nf									
35	2-Ethyl hexanal	41 [27]	Beany [27]			0.004	0.004	0.005	0.004	0.007	0.010	0.009
36	3-(Methylthio)-nonanal	Nf	Nf									
37	(E)-2-nonenal	0.00019 [28]	fatty, tallow, beans, cucumber, woody-like [59]		457.89	984.21	926.32	710.53	436.84	652.63	778.95	736.84
38	2,2-Dimethyl propanal	Nf	Nf									
39	(E)-2-Hexenal	0.11 [28]	fresh, fruity, green-like, sweet [59]	2.67								
40	5-Methyl-2-furancarboxaldehyde	Nf	Nf									
41	Hexanoic acid (caproic)	3.0 [28]	sweet, cheesy [59]		0.28	0.39	0.48	0.86	1.49	1.12	0.67	0.59
42	Octanoic acid (caprylic)	8.8 [28]	sweet, cheesy [59]		0.06	0.14	0.21	0.19	0.55	0.39	0.23	0.27
43	Nonanoic acid (pelargonic)	4.6–9.0 [28]	fatty-like [59]			0.01 *	0.02 *	0.01 *	0.02 *	0.03 *	0.04 *	0.04 *
44	Acetic acid	99 [28]	vinegar, peppers, green, fruity, floral, sour [72]	0.01	0.01	0.01	0.02	0.04	0.01	0.01	0.02	0.02
45	Pentanoic acid	11 [28]	sweaty, fruity [65]		0.01	0.02	0.02	0.03	0.02	0.04	0.04	0.03
46	4-Methylpentanoic acid	0.81 [28]	Sweaty [73]	0.14								
47	2-Methylhexanoic acid	0.92–2.70 [28]	sweat, oily [74]		0.09 *	0.19 *	0.12 *	0.09 *	0.33 *	0.34 *	0.35 *	0.29 *
48	3,3-Dimethyl-2-butanone	Nf	Nf									
49	4-Methyl-2-hexanone	0.00081–0.0041 [28]	Fruity [75]	19.55 *	19.15 *	19.55 *	20.37 *	19.55 *	17.11 *	17.92 *	22.81 *	19.55 *
50	3-Hexanone	0.041–0.081 [28]	ethereal, grape [76]	2.57 *	2.66 *	2.33 *	2.71 *	2.67 *	1.90 *	2.34 *	2.00 *	1.88 *
51	2,6-Dimethyl-4-heptanone	0.11 [29]	fruity, sweet [77]	2.50	6.04	6.11	6.49	6.99	4.29	5.12	3.54	4.69
52	5-Dodecanone	Nf	Nf									
53	Dyhidroyiacetone	Nf	Nf									
54	1-Acetyloxy-2-propanone	Nf	Nf									
55	3-Butyldihydro-2(3H)-furanone	Nf	Nf									
56	5-Methyl-2(3H)-furanone	Nf	Nf									
57	Tetrahydro-2,5-dimethyl-furan	Nf	Nf									
58	1-Butoxy-3-methyl-2-butene	Nf	Nf									
59	Ethyl-1-propenyl ether	Nf	Nf									
60	2,4-Dimethyl-2-pentene	Nf	Nf									
61	(Z)-3-Dodecene	Nf	Nf									
62	4-Cyclopenten-1,3-dione	Nf	Nf									
63	1,2,3-Trimethyl-benzene	Nf	Nf									
64	Butylhydroxytoluene	Nf	Nf									
65	Trichloromethane	0.12 [28]	pleasant, etheric, nonirritating [78]	2.29					2.69			2.65
66	3-Trifluoroacetoxy dodecane	Nf	Nf									
67	1-Hydroperoxyhexane	Nf	Nf									
68	Cis-2-methyl-3-propyl-oxirane	Nf	Nf									

Nf: not found—* Calculated using the mean ODT.

## Data Availability

Data is contained within the article.

## References

[1] Cais-Sokolińska D., Wójtowski J., Pikul J. (2016). Rheological, texture and sensory properties of kefir from mare’s milk and its mixtures with goat and sheep milk. Mljekarstvo/Dairy.

[2] Farnworth E.R., Mainville I., Farnworth E.R. (2008). Kefir−A fermented milk product. Handbook of Fermented Functional Foods.

[3] Abraham A., De Antoni G. (1999). Characteristics of kefir grain grown in milk and in soy milk. J. Dairy Res..

[4] Piermaria J.A., de la Canal M.L., Abraham A.G. (2008). Gelling properties of kefiran, a food-grade polysaccharide obtained from kefir grain. Food Hydrocoll..

[5] Gösta M., López A. (2003). Manual de Industrias Lácteas. Tetra Pak AB Processing Systems: Madrid, Spain.

[6] Fiorda F.A., Pereira G.V.M., Thomaz-Soccol V., Medeiros A.P., Rakshit S.K., Soccol C.R. (2016). Development of kefir-based probiotic beverages with DNA protection and antioxidant activities using soybean hydrolyzed extract, colostrum and honey. LWT -Food Sci. Technol..

[7] Hertzler S.R., Clancy S.M. (2003). Kefir improves lactose digestion and tolerance in adults with lactose maldigestion. J. Am. Diet. Assoc..

[8] Liu J.R., Wang S.Y., Chen M.J., Chen H.L., Yueh P.Y., Lin C.W. (2006). Hypocholesterolaemic effects of milk-kefir and soyamilk-kefir in cholesterol-fed hamsters. Br. J. Nut..

[9] Ismaiel A.A., Ghaly M.F., El-Naggar A.K. (2011). Some physicochemical analyses of kefir produced under different fermentation conditions. J. Sci. Ind. Res..

[10] Puerari C., Magalhães K.T., Schwan R.F. (2012). New cocoa pulp-based kefir beverages: Microbiological, chemical composition and sensory analysis. Food Res. Int..

[11] Corona O., Randazzo W., Miceli A., Guarcello R., Francesca N., Erten H., Moschetti G., Settanni L. (2016). Characterization of kefir-like beverages produced from vegetable juices. LWT-Food Sci. Technol..

[12] Randazzo W., Corona O., Guarcello R., Francesca N., Germanà M.A., Erten H., Moschetti G., Settanni L. (2016). Development of new non-dairy beverages from Mediterranean fruit juices fermented with water kefir microorganisms. Food Microbiol..

[13] Shanmuganayagam D., Warner T.F., Krueger C.G., Reed J.D., Folts J.D. (2007). Concord grape juice attenuates platelet aggregation, serum cholesterol and development of atheroma in hypercholesterolemic rabbits. Atherosclerosis.

[14] God J.M., Tate P., Larcom L.L. (2007). Anticancer effects of four varieties of muscadine grape. J. Med. Food.

[15] Jung K., Wallig M., Singletary K. (2006). Purple grape juice inhibits 7,12-dimethylbenz-[a]anthracene (DMBA)-induced rat mammary tumorigenesis and in vivo DMBA-DNA adduct formation. Cancer Lett..

[16] Meyer A.S., Yi O.S., Pearson D.A., Waterhouse A.L., Frankel E.N. (1997). Inhibition of human low density lipoprotein oxidation in relation to composition of phenolic antioxidants in grapes (*Vitis vinifera*). J. Agric. Food Chem..

[17] Rodriguez-Vaquero M.J., Alberto M.R., Manca-de-Nadra M.C. (2007). Antibacterial effect of phenolic compounds from different wines. Food Cont..

[18] Piva C.R., Garcia J.L.L., Morgan W. (2006). The ideal table grapes for the Spanish market. Rev. Bras. Frutic..

[19] Costas M., Alonso E., Guerra N.P. (2016). Nisin production in realkalized fed-batch cultures in whey with feeding with lactose- or glucose-containing substrates. Appl. Microbiol. Biotechnol..

[20] Costas M., Alonso E., Outeiriño D., Fajardo P., Guerra N.P. (2017). Combination of food wastes for an efficient production of nisin in realkalized fed-batch cultures. Biochem. Eng. J..

[21] Poolman B., Konings W.N. (1988). Relation of growth of *Streptococcus lactis* and *Streptococcus cremoris* to amino acid transport. J. Bacteriol..

[22] Fajardo P., Rodríguez I., Pastrana L., Guerra N.P. (2008). Production of a potentially probiotic culture of *Lactobacillus casei* subsp. *casei* CECT 4043 in whey. Int. Dairy J..

[23] Chen X.H., Lou W.Y., Zong M.H., Smith T.J. (2011). Optimization of culture conditions to produce high yields of active *Acetobacter* sp. CCTCC M209061 cells for anti-prelog reduction of prochiral ketones. BMC Biotechnol..

[24] Membré J.M., Kubaczka M., Chéné C. (1999). Combined effects of pH and sugar on growth rate of *Zygosaccharomyces rouxii*, a bakery product spoilage yeast. Appl. Environ. Microbiol..

[25] Arroyo-López F.N., Orlić S., Querol A., Barrio E. (2009). Effects of temperature, pH and sugar concentration on the growth parameters of *Saccharomyces cerevisiae*, *S. kudriavzevii* and their interspecific hybrid. Int. J. Food Microbiol..

[26] Bartowsky E.J., Xia D., Gibson R.L., Fleet G.H., Henschke P.A. (2003). Spoilage of bottled red wine by acetic acid bacteria. Lett. Appl. Microbiol..

[27] Burdock G.A. (2010). Fenaroli’s Handbook of Flavour Ingredients.

[28] Van Gemert L.J. (2011). Odour Thresholds. Compilations of Odour Threshold Values in Air, Water and Other Media.

[29] Bingham E., Cohrssen B., Powell C.H. (2001). Patty’s Toxicology.

[30] Pino J.A., Quijano C.E. (2012). Study of the volatile compounds from plum (*Prunus domestica* L. cv. Horvin) and estimation of their contribution to the fruit aroma. Ciência Tecnol. Aliment..

[31] Cheirsilp B., Shimizu H., Shioya S. (2003). Enhanced kefiran production of *Lactobacillus kefiranofaciens* by mixed culture with *Saccharomyces cerevisiae*. J. Biotechnol..

[32] Oude S.J.W.H., Krooneman J., Gottschal J.C., Spoelstra S.F., Faber F., Driehuis F. (2001). Anaerobic conversion of lactic acid to acetic acid and 1,2-propanediol by *Lactobacillus buchneri*. Appl. Environ. Microbiol..

[33] Cheirsilp B., Shoji H., Shimizu H., Shioya S. (2003). Interactions between *Lactobacillus kefiranofaciens* and *Saccharomyces cerevisiae* in mixed culture for kefiran production. J. Biosci. Bioeng..

[34] Cheirsilp B., Radchabut S. (2011). Use of whey lactose from dairy industry for economical kefiran production by *Lactobacillus kefiranofaciens* in mixed cultures with yeasts. New Biotechnol..

[35] Felipe M.G., Vieira D.C., Vitolo M., Silva S.S., Roberto I.C., Manchilha I.M. (1995). Effect of acetic acid on xylose fermentation to xylitol by *Candida guilliermondii*. J. Basic Microbiol..

[36] Kirtadze E., Nutsubidze N. (2009). Metabolic potential of alcoholic fermentation yeasts. Bull. Georgian Natl. Acad. Sci..

[37] Mendes A., Mendes-Faia A. (2020). The role of yeasts and lactic acid bacteria on the metabolism of organic acids during winemaking. Foods.

[38] Yalçin S.K., Özbaş Z.Y. (2005). Determination of growth and glycerol production kinetics of a wine yeast strain *Saccharomyces cerevisiae* Kalecik 1 in different substrate media. World J. Microbiol. Biotechnol..

[39] Azhar S.H.M., Abdulla R., Jambo S.A., Marbawi H., Gansau J.A., Faik A.A.M., Rodrigues K.F. (2017). Yeasts in sustainable bioethanol production: A review. Biochem. Biophys. Rep..

[40] Lonvaud-Funel A. (1999). Lactic acid bacteria in the quality improvement and depreciation of wine. Antonie Leeuwenhoek.

[41] Reale A., Di Renzo T., Rossi F., Zotta T., Iacumin L., Preziuso M., Parente E., Sorrentino E., Coppol R. (2015). Tolerance of *Lactobacillus casei*, *Lactobacillus paracasei* and *Lactobacillus rhamnosus* strains to stress factors encountered in food processing and in the gastro-intestinal tract. LWT-Food Sci. Technol..

[42] Sánchez C., Neves A.R., Cavalheiro J., dos Santos M.M., García-Quintáns N., López P., Santos H. (2008). Contribution of citrate metabolism to the growth of *Lactococcus lactis* CRL264 at low pH. Appl. Environ. Microbiol..

[43] Rimada P.S., Abraham A.G. (2001). Polysaccharide production by kefir grains during whey fermentation. J. Dairy Res..

[44] Maeda H., Zhu X., Suzuki S., Suzuki K., Kitamura S. (2004). Structural characterization and biological activities of an exopolysaccharide kefiran produced by *Lactobacillus kefiranofaciens* WT-2B^T^. J. Agric. Food Chem..

[45] Dailin D.J., Elsayed A.E., Othman N.Z., Malek R., Phin H.S., Aziz R., Wadaan M., El Enshasy H.A. (2016). Bioprocess development for kefiran production by *Lactobacillus kefiranofaciens* in semi industrial scale bioreactor. Saudi J. Biol. Sci..

[46] Gradova N.B., Khokhlacheva A.A., Murzina E.D., Myasoyedova V.V. (2015). Microbial components of kefir grains as exopolysaccharide kefiran producers. Appl. Biochem. Microbiol..

[47] (2011). Regulation (EU) No. 1169/2011 of the European Parliament and of the Council of October 25, 2011 on the provision of food information to consumers, amending Regulations (EC) No 1924/2006 and (EC) No 1925/2006 of the European Parliament and of the Council, and repealing Commission Directive 87/250/EEC, Council Directive 90/496/EEC, Commission Directive 1999/10/EC, Directive 2000/13/EC of the European Parliament and of the Council, Commission Directives 2002/67/EC and 2008/5/EC and Commission Regulation (EC) No 608/2004. Off. J. Eur. Union.

[48] Viana R.O., Teixeira K., Braga R.A., Dias D.R., Schwan R.F. (2017). Fermentation process for production of apple-based kefir vinegar: Microbiological, chemical and sensory analysis. Braz. J. Microbiol..

[49] Garde T., Lorenzo C., Carot J.M., Esteve M.D., Climent M.D., Salinas M.R. (2009). Differentiation of barrel-aged wines according to their origin, variety, storage time and enological parameters using fermentation products. Food Cont..

[50] Sánchez-Palomo E., Izquierdo Cañas P.M., Delgado J.A., Viñas M.A.G. (2018). Sensory characterization of wines obtained by blending cencibel grapes and minority grape varieties cultivated in La Mancha region. J. Food Qual..

[51] Vilanova M., Genisheva Z., Graña M., Oliveira J.M. (2013). Determination of odorants in varietal wines from international grape cultivars (*Vitis vinifera*) grown in NW Spain. S. Afr. J. Enol. Vitic..

[52] Vilanova M., Freire L. (2017). Complementary effect of blending on the volatile composition of albariño and loureira white wines (*Vitis vinifera* L.). S. Afr. J. Enol. Vitic..

[53] Dragone G., Mussatto S.I., Oliveira J.M., Teixeira J.A. (2009). Characterisation of volatile compounds in an alcoholic beverage produced by whey fermentation. Food Chem..

[54] Alonso E., Torrado A., Pastrana L., Orriols I., Pérez-Guerra N. (2010). Production and characterization of distilled alcoholic beverages obtained by solid-state fermentation of black mulberry (*Morus nigra* L.) and black currant (*Ribes nigrum* L.). J. Agric. Food Chem..

[55] Cortés S., de la Peña M.L.G., Gómez E.F. (2005). Volatile composition and sensory characters of commercial Galician orujo spirits. J. Agric. Food Chem..

[56] Garcia-Carpintero E.G., Sanchez-Palomo E., Gallego M.A.G., Gonzalez-Viñas M.A. (2011). Volatile and sensory characterization of red wines from cv. *Moravia Agriaminority* grape variety cultivated in La Mancha region over five consecutive vintages. Food Res. Int..

[57] Cortés S., Rodriguez R., Domínguez J.M., Díaz E. (2015). Impact odorants and sensory profile of young red wines from four Galician (NW of Spain) traditional cultivars. J. Inst. Brew..

[58] Ferreira V., Culleré L., López R., Cacho J. (2004). Determination of important odor-active aldehydes of wine through gas chromatography–mass spectrometry of their O-(2,3,4,5,6-pentafluorobenzyl)oximes formed directly in the solid phase extraction cartridge used for selective isolation. J. Chromatogr. A..

[59] Verma D.K., Srivastav P.P. (2020). A paradigm of volatile aroma compounds in rice and their product with extraction and identification methods: A comprehensive review. Food Res. Int..

[60] Duarte W.F., Dias D.R., Oliveira J.M., Teixeira J.A., de Almeida e Silva J.B., Schwan R.F. (2010). Characterization of different fruit wines made from cacao, cupuassu, gabiroba, jaboticaba and umbu. LWT - Food Sci. Technol..

[61] Perestrelo R., Fernandes A., Albuquerque F.F., Marques J.C., Câmara J.S. (2006). Analytical characterization of the aroma of Tinta Negra Mole red wine: Identification of the main odorants compounds. Anal. Chim. Acta.

[62] Ferreira V., Reynolds A.G. (2010). Volatile aroma compounds and wine sensory attributes. Managing Wine Quality. Viticulture and Wine Quality.

[63] Samappito S., Butkhup L. (2010). Effect of skin contact treatments on the aroma profile and chemical components of mulberry (*Morus alba* Linn.) wines. Afr. J. Food Sci..

[64] Mei J., Liu F., Fang S., Lan W., Xie J. (2020). High-CO_2_ modified atmosphere packaging with superchilling (−1.3 °C) inhibit biochemical and flavor changes in turbot (*Scophthalmus maximus*) during storage. Molecules.

[65] Czerny M., Christlbauer M., Christlbauer M., Fischer A., Granvogl M., Hammer M., Hartl C., Moran N., Schieberle P. (2008). Re-investigation on odour thresholds of key food aroma compounds and development of an aroma language based on odour qualities of defined aqueous odorant solutions. Eur. Food Res. Technol..

[66] De Sousa M., Narain N., do Socorro M., Nunes M.L. (2011). Volatile compounds and descriptive odor attributes in umbu (*Spondias tuberosa*) fruits during maturation. Food Res. Int..

[67] Arcari S.G., Caliari V., Sganzerla M., Godoy H.T. (2017). Volatile composition of Merlot red wine and its contribution to the aroma: Optimization and validation of analytical method. Talanta.

[68] Welke J.E., Zanus M., Lazzarotto M., Zini C.A. (2014). Quantitative analysis of headspace volatile compounds using comprehensive two-dimensional gas chromatography and their contribution to the aroma of Chardonnay wine. Food Res. Int..

[69] Liu S.Q., Holland R., Crow V.L. (2004). Esters and their biosynthesis in fermented dairy products: A review. Int. Dairy J..

[70] Zhao P., Gao J., Qian M., Li H. (2017). Characterization of the key aroma compounds in chinese syrah wine by gas chromatography-olfactometry-mass spectrometry and aroma reconstitution studies. Molecules.

[71] Bowen A.J., Reynolds A.G. (2012). Odor potency of aroma compounds in Riesling and Vidal blanc table wines and icewines by gas chromatography−olfactometry−mass spectrometry. J. Agric. Food Chem..

[72] Walsh A.M., Crispie F., Kilcawley K., O’Sullivan O., O’Sullivan M.G., Claesson M.J., Cotter P.D. (2016). Microbial succession and flavor production in the fermented dairy beverage kefir. MSystems.

[73] Zhai X., Granvogl M. (2019). Characterization of the key aroma compounds in two differently dried *Toona sinensis* (A. Juss.) Roem. by means of the molecular sensory science concept. J. Agric. Food Chem..

[74] Brennand C.P., Ha J.K., Lindsay R.C. (1989). Aroma properties and thresholds of some branched-chain and other minor volatile fatty acids occurring in milkfat and meat lipids. J. Sens. Stud..

[75] Reale A., Di Renzo T., Boscaino F., Nazzaro F., Fratianni F., Aponte M. (2019). Lactic acid bacteria biota and aroma profile of Italian traditional sourdoughs from the irpinian area in Italy. Front. Microbiol..

[76] Barron L.J.R., Redondo Y., Aramburu M., Perez-Elortondo F.J., Albisu M., Najera A.I., de Renobales M. (2005). Variations in volatile compounds and flavor in Idiazabal cheese manufactured from ewe’s milk in farmhouse and factory. J. Sci. Food. Agric..

[77] Dertli E., Çon A.H. (2017). Microbial diversity of traditional kefir grains and their role on kefir aroma. LWT -Food Sci. Technol..

[78] Prager J.C., Prager J.C. (1995). Environmental Contaminant Reference Databook.

